# Low-density lipoprotein receptor-related protein 1 (LRP1) is a novel receptor for apolipoprotein A4 (APOA4) in adipose tissue

**DOI:** 10.1038/s41598-021-92711-0

**Published:** 2021-06-24

**Authors:** Jie Qu, Sarah Fourman, Maureen Fitzgerald, Min Liu, Supna Nair, Juan Oses-Prieto, Alma Burlingame, John H. Morris, W. Sean Davidson, Patrick Tso, Aditi Bhargava

**Affiliations:** 1 Department of Pathology and Laboratory Medicine, University of Cincinnati College of Medicine, 2180 E Galbraith Road, Cincinnati, 45237-0507 USA; 2 Departments of Pharmaceutical Chemistry, University of California San Francisco, 600 16th Street, San Francisco, CA 94158 USA; 3 Department of Obstetrics and Gynecology, Center for Reproductive Sciences, University of California San Francisco, 513 Parnassus Avenue, Rm HSE1636, San Francisco, CA 94143-0556 USA

**Keywords:** Diseases, Endocrinology, Molecular medicine

## Abstract

Apolipoprotein A4 (APOA4) is one of the most abundant and versatile apolipoproteins facilitating lipid transport and metabolism. APOA4 is synthesized in the small intestine, packaged onto chylomicrons, secreted into intestinal lymph and transported via circulation to several tissues, including adipose. Since its discovery nearly 4 decades ago, to date, only platelet integrin αIIbβ3 has been identified as APOA4 receptor in the plasma. Using co-immunoprecipitation coupled with mass spectrometry, we probed the APOA4 interactome in mouse gonadal fat tissue, where *ApoA4* gene is not transcribed but APOA4 protein is abundant. We demonstrate that lipoprotein receptor-related protein 1 (LRP1) is the cognate receptor for APOA4 in adipose tissue. LRP1 colocalized with APOA4 in adipocytes; it interacted with APOA4 under fasting condition and their interaction was enhanced during lipid feeding concomitant with increased APOA4 levels in plasma. In 3T3-L1 mature adipocytes, APOA4 promoted glucose uptake both in absence and presence of insulin in a dose-dependent manner. Knockdown of LRP1 abrogated APOA4-induced glucose uptake as well as activation of phosphatidylinositol 3 kinase (PI3K)-mediated protein kinase B (AKT). Taken together, we identified LRP1 as a novel receptor for APOA4 in promoting glucose uptake. Considering both APOA4 and LRP1 are multifunctional players in lipid and glucose metabolism, our finding opens up a door to better understand the molecular mechanisms along APOA4-LRP1 axis, whose dysregulation leads to obesity, cardiovascular disease, and diabetes.

## Introduction

Metabolic syndrome, a cluster of conditions that increase the risks of obesity and type 2 diabetes, together with cardiovascular diseases has reached epidemic proportions worldwide in the past decade^1^. The underlying causes for metabolic syndrome include dysregulation of lipid metabolism and glucose homeostasis, such as dyslipidemia or insulin resistance^2–4^. The human apolipoprotein *(APO) A4* gene is located in the *APOA1/C3/A4/A5* gene cluster on chromosome 11q23 and the mouse *ApoA4* gene is on chromosome 9. This gene cluster is essential for lipoprotein metabolism, lipid homeostasis and susceptibility to cardiovascular diseases^5–7^. APOA4, the protein product of *ApoA4*, is primarily synthesized in mammalian intestine and a small portion in rodent liver and brain^8–11^. Among these apolipoprotein clusters, APOA4 is unique in that intestinal *ApoA4* gene expression is rapidly induced by fat absorption^12^, and APOA4 protein is an abundant circulating apolipoprotein, as determined in the plasma of humans (~ 15 mg/dL) and rats (~ 24 mg/dL)^13^. Upon fat absorption, APOA4 produced by the enterocytes of the small intestine is incorporated into triglyceride (TG)-rich chylomicrons and secreted into intestinal lymph. As the lipolysis of TG occurs, APOA4 dissociates from chylomicron remnants, transfers to high-density lipoproteins or circulates in lipoprotein-free form in the blood^14^. In addition to facilitating fatty acid uptake and lipid metabolism^15–18^, APOA4 is involved in various aspects of glucose homeostasis, including inhibition of gluconeogenesis in hepatocytes^19,20^, promotion of glucose uptake in adipocytes^21^ and enhancement of insulin secretion in pancreatic islets^22^, but the receptors that mediate these actions of APOA4 have remained unidentified. Moreover, APOA4 protects against inflammation and atherosclerosis^23^, prevents platelet aggregation, plaque disruption and thrombosis^24,25^ in the periphery, and inhibits food intake through central nervous system^26^. In clinical studies, plasma APOA4 levels are inversely related to coronary artery disease^27^. APOA4 levels increase several folds^28^ after Roux-en-Y gastric bypass surgery, which improves satiety and obesity-related comorbidities in patients, and positively correlate with improved insulin sensitivity^29^. APOA4 is proposed as an early diagnostic biomarker for prediabetes, liver fibrosis, and impaired renal function^30–32^. Genome-wide association studies have revealed that an *APOA4* single nucleotide polymorphism (SNP), rs5104, is associated with dyslipidemia in Han Chinese and with triglycerides in response to lipid-lowering treatment drug, fenofibrate^6,33^.

Low-density lipoprotein receptor related protein 1 (LRP1) is a multifunctional transmembrane receptor with endocytic and signaling properties^34^. LRP1 is composed of a 515-kDa ligand-binding α-subunit, and an 85-kDa transmembrane and intracellular β-subunit^35^. *Lrp1* gene is ubiquitously expressed in several tissues, including hepatocytes, adipocytes, neurons, vascular smooth muscle cells, fibroblasts and macrophages. In the liver, LRP1 plays prominent role in lipid homeostasis by mediating the clearance of ApoE-enriched chylomicron and very low-density lipoprotein remnants^36^ and by interacting with hepatic lipase and lipoprotein lipase^37^. LRP1 is also important for glucose metabolism by suppressing gluconeogenesis and protecting against hepatic insulin resistance^38^. In adipose tissue, it regulates postprandial lipid clearance, glucose metabolism and energy homeostasis^39^. In the brain, it modulates glucose metabolism and insulin signaling^40^. Additionally, it functions to remove excessive amyloid-β peptides^41,42^, whose deposition is one of the major histological hallmarks of Alzheimer’s Disease. In fibroblasts and smooth muscle cells, LRP1 potentiates growth factor signaling pathways, such as platelet-derived growth factor receptor β (PDGFRβ), to activate cell proliferation and regulate vascular wall integrity^43,44^. In macrophages, LRP1 plays anti-inflammatory role to prevent atherosclerosis^45,46^. Genome-wide association studies have shown that LRP1 SNPs are associate with coronary heart disease^47^, abdominal aortic aneurysm^48,49^, Alzheimer’s Disease^50^, venous thrombosis^51^, metabolic syndrome prevalence^52^, and carbohydrate metabolism in subjects with the metabolic syndrome^53^.

Although much has been learned about the physiological functions of APOA4 over the past 40 years, APOA4 interacting proteins are poorly understood. To date, only platelet αIIbβ3 integrin^24^, nuclear receptor subfamily 1 group D member 1 (NR1D1) and nuclear receptor subfamily 4 group A member 1 (NR4A1) have been shown to physically interact with APOA4^19,20^. Human APOA4 binds to the cell surface of activated platelet via αIIbβ3 integrin and negatively regulates αIIbβ3-mediated platelet aggregation and thrombosis^24^. Therefore, αIIbβ3 integrin can be considered as APOA4 receptor in the plasma. NR1D1/NR4A1 interaction with APOA4 occurs exclusively in the cytoplasm and nucleus of cultured HepG2 cells^19,20^. Thus, they may not act as APOA4 receptor because exogenous APOA4 has to somehow be internalized by hepatocytes prior to its interaction with NR1D1 or NR4A1 intracellularly. APOA4 has also been reported to bind to cell surfaces of several cell lines, including adult bovine aortic endothelial cells^54^, mouse adipose cells^55^ and primary rat hepatocytes^56^. However, the membrane proteins interacting with APOA4 remain largely unknown.

Given that both human and mouse APOA4 bind mouse adipocytes and mouse APOA4 promoted glucose uptake in adipocytes, here, we probed adipocyte-specific APOA4 interactome by co-immunoprecipitation (co-IP) followed by mass spectrometry (MS). Although *ApoA4* gene is active predominantly in the rodent intestine, surprisingly, we found abundant APOA4 in gonadal adipose tissues, suggesting rapid uptake of APOA4 by adipocytes from circulation. In this study, we identified LRP1 as an APOA4 interacting protein in vivo, where the two proteins robustly co-localized in adipocytes. We demonstrated that APOA4 and LRP1 protein levels increased in-step after lipid feeding and that LRP1 is a cognate receptor for APOA4 in adipocytes that accounts for APOA4- and insulin-stimulated glucose uptake as well as PI3K-AKT signaling in adipocytes.

## Results

### Uptake of APOA4 in adipose tissue from circulation

**Figure 1:**
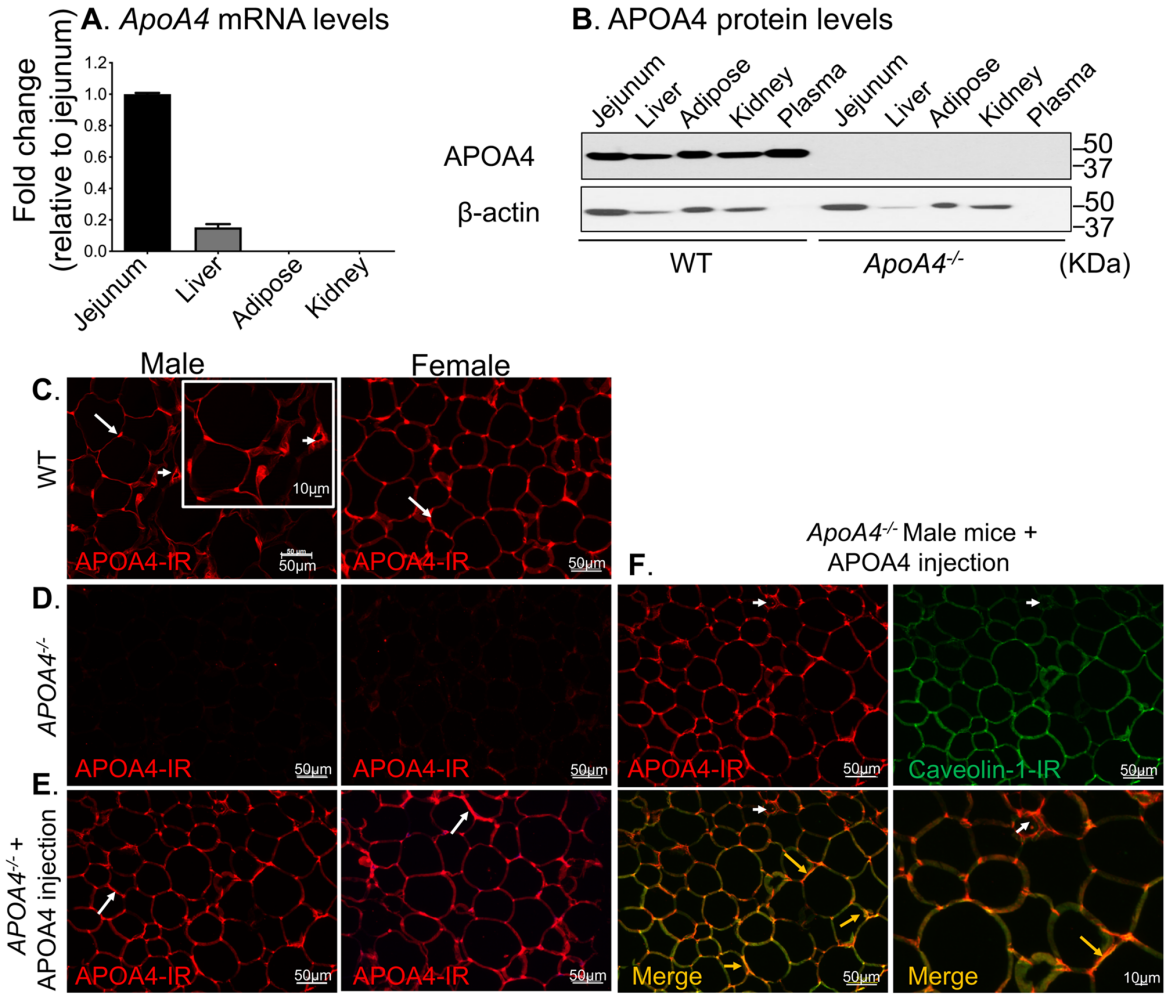
*APOA4* is transcribed and translated primarily in the gut and transported to adipose tissue via circulation. **(A)** *APOA4* gene was expressed in mouse jejunum and liver tissues, but not in gonadal adipose tissue and kidney. RT-PCR for *ApoA4* and *β-actin* was performed on total mRNA isolated from jejunum, liver, gonadal fat, and kidney tissues from male and female 129X1/SvJ WT mice (n = 3 for each sex). *ApoA4* levels were normalized with *β-actin* levels and data was presented as fold change over normalized *ApoA4* mRNA levels in jejunum. See Supplemental Fig. S1. **(B)** Presence of APOA4 protein in tissues of WT but not of *ApoA4*^*−/−*^ mice. Total protein (40 µg) from jejunum, liver, epididymal fat and kidney tissues and plasma (10 μg) were separated by SDS-PAGE and probed with anti-APOA4 antibody. The blot was stripped and probed with anti-β-actin for loading control and used for normalization. A representative western blot image shows the presence of APOA4 protein in all tissues from WT, but not *ApoA4*^*−/−*^ mice (n = 3/group); these findings also confirmed specificity of the antibody used. **(C-E)** Representative immunofluorescence confocal images of APOA4 immunoreactivity (IR) in mouse gonadal fat tissues. Epididymal fat from male and periovarian fat from female mice were harvested, fixed, paraffin embedded, sectioned and stained with anti-APOA4 and anti-caveolin-1 (n = 2 technical and 3 biological replicates). Scale bar: 50 μm. **(C)** APOA4 immunoreactivity (APOA4-IR) was present at the cell surface of adipocytes in a discrete manner in male and female WT mice (arrows) and in the vasculature (inset: arrowhead), **(D)** no APOA4-IR was detected in *ApoA4*^*−/−*^ mice, thereby also confirming specificity of the antibody used. **(E)** I.p. injection of r-m-APOA4 (5 μg/g body weight) in *ApoA4*^*−/−*^ mice resulted in APOA4 uptake in gonadal fat tissues with similar pattern of localization of APOA4-IR in adipocytes as that in WT mice (arrows show discrete cell surface expression). **(F)** Apo-A-IV-IR co-localized with the adipocyte cell surface marker caveolin-1 (Merge, yellow arrows), but not in the vasculature (white arrowhead), confirming that exogenous APOA4 is targeted to the appropriate cell types. Scale bar: 50 μm and 10 µm.

Mammalian APOA4 protein is abundantly and ubiquitously expressed in several tissues but transcription from *ApoA4* gene is restricted predominantly to a few organs, including the small intestine^57,58^. Here, we found the highest levels of *ApoA4* mRNA levels in the jejunum; only about 15% mRNA transcripts were found in the liver relative to the jejunum. Importantly, no amplifiable transcripts were detected in the gonadal (either epididymal or periovarian) adipose tissues or kidney of wildtype (WT, 129X1/SvJ strain) male and female mice (Fig. 1A). Next, we reasoned that APOA4 protein synthesized in the gut and/or the liver is transported via circulation to target organs that do not normally transcribe *ApoA4* mRNA. Immunoblotting confirmed presence of APOA4 protein in the plasma (circulation) as well as in the adipose and kidney tissues of WT, but not global *ApoA4*^*−/−*^ mice (Fig. 1B). A single band at ~ 43 kDa corresponding to mature APOA4 present in the tissues and plasma of WT, but not *ApoA4*^*−/−*^ mice indicated the specificity of APOA4 antibodies used. Immunostaining and confocal microscopy confirmed the presence of immunoreactive APOA4 in gonadal adipose tissues from both sexes of WT mice, where it localized to the cell surface and in the vasculature (Fig. 1C, arrow and inset: arrowhead, respectively), but no immunoreactivity was detected in gonadal adipose tissue of *ApoA4*^*−/−*^ mice (Fig. 1D). APOA4 was also localized on the cell surface of adipocytes from subcutaneous and mesenteric fat pads from WT mice (see Supplementary Fig. S1 online). To further confirm that APOA4 reaches the gonadal adipose tissue via circulation, we administered a bolus of recombinant mouse APOA4 (r-m-APOA4) intraperitoneally (i.p.) in *ApoA4*^*−/−*^ mice. Two-hours after administration of r-m-APOA4, immunostaining and confocal microscopy revealed presence of APOA4 in target tissues and localized to the cell membrane and in discrete puncta of epididymal adipose tissue of *ApoA4*^*−/−*^ mice in a pattern similar to that observed in WT mice (Fig. 1E, arrows). Furthermore, APOA4 colocalized with caveolin-1, a highly expressed cell surface protein in adipocytes^59–61^ (Fig. 1F, yellow arrows); importantly, no caveolin-1 was detectable in the vasculature but APOA4 immunoreactivity was clearly present in the vasculature (Fig. 1F, Merge, white arrowhead), further suggesting that APOA4 reaches the gonadal adipose tissues via circulation.

### Identification of LRP1 as a novel APOA4-interacting protein by probing the APOA4 interactome using mass spectrometry

**Figure 2:**
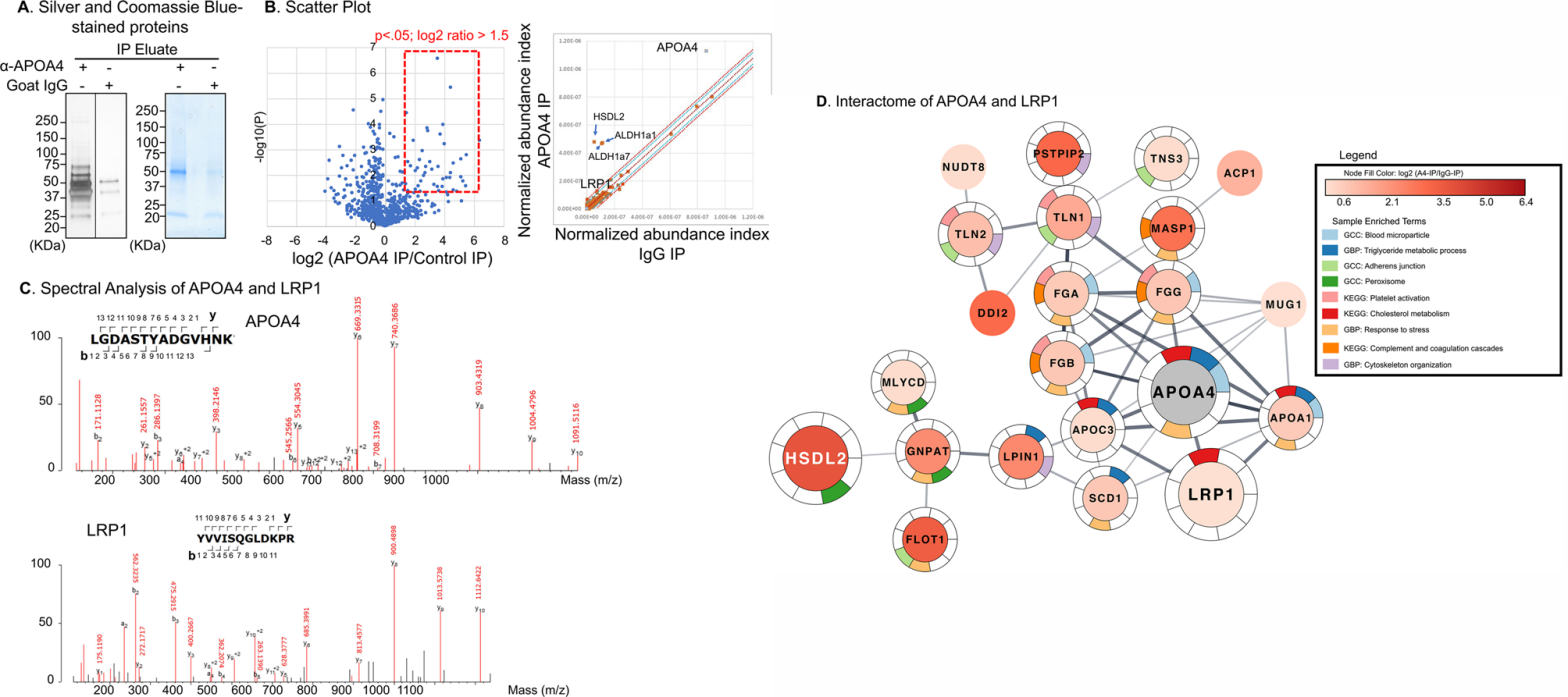
Identification of APOA4 interactome in mouse gonadal fat tissues using mass spectrometry. Wildtype 129X1/SvJ male and female mice fasted for 5 h and i.p. injected with a single dose of r-m-APOA4 (2.5 μg/g body weight). Two hours post injection, mice were euthanized and total protein was isolated from the epididymal and periovarian fat tissues (n = 5–6/sex). IP was performed from pooled samples (10 mg total protein per IP experiment) using anti(α)-APOA4-specific and goat IgG (negative control) antibodies in parallel pull-down experiments. Protein eluates were electrophoresed on SDS-PAGE and visualized by (**A**) Silver and Coomassie blue–stained gel of IPs showed several bands enriched in α-APOA4 pull-downs, but not in goat IgG. Silver-stained lanes from the same gel are shown. α-APOA4 and IgG lanes were excised and processed for mass spectrometry. (*n* = 5–6/condition for IP and 4/condition for MS). (**B**) Volcano plot showing relative abundances of the proteins found in APOA4 Co-IP experiments. X axis shows the log 2 of the relative abundances over IgG control pull downs. Y axis the significance as – log_10_ of P values. The proteins that lie within the red thick dotted line in the volcano plot were plotted in network generated in (**D**). The right panel shows data as scatter plot of relative enrichment of APOA4-associated proteins vs. IgG pull-downs. Intervals of confidence for 95% (blue lines) and 99.7% (red lines) are indicated. *HSDL2* hydroxysteroid dehydrogenase like 2; *ALDH1a1/a7* aldehyde dehydrogenase 1 family members A1and A7. (**C**) Spectral analysis: High-energy collision dissociation–tandem mass spectra obtained from precursor ions with mass 483.2320^+3^ (APOA4) and 687.8901^+2^ (LRP1) found in tryptic digests of immunoaffinity pull-downs of anti-APOA4 corresponded to peptides spanning residues L66 to K79 and Y2001 to R2012 of APOA4 and LRP1, respectively. b- and y-type ion series are labeled. (**D**) Portion of the STRING interactome in the neighborhood around APOA4 highlighted within the red box in (**B**). The nodes are colored by the Log2 ratio of APOA4 Co-IP/IgG control, except for APOA4, which is colored grey, and the edge widths represent the STRING confidence score. The colored slices around the outside of the nodes represent the enrichment as provided by the Cytoscape stringApp, filtering for only KEGG Pathways (KEGG), GO Cellular Component (GCC), and GO Biologic Process (GBP). The nodes HSDL2, LRP1, and APOA4 are enlarged to correspond with highlighted nodes in the scatter plot shown in 2B. The label color for HSDL2 was changed to white to enhance visibility.

Identification of APOA4 receptors in the adipose tissues has been elusive. Since APOA4 is primarily synthesized in the gut and transported via the circulation to several target tissues, we suspected APOA4 may have target tissue-specific interacting partners and receptors. In order to identify adipose-specific APOA4-interacting proteins, r-m-APOA4 was administered i.p. in male and female WT mice fasted for 5 h and immunoprecipitation from adipose tissue lysates was performed 2 h-post injection. Pull down with goat anti (α)-APOA4 or goat IgG were performed in parallel. Eluted co-immunoprecipitated proteins were separated by SDS-PAGE and visualized by silver and Coomassie blue stains (Fig. 2A). Several proteins were enriched in α-APOA4 versus IgG pull downs (Fig. 2B–D); individual lanes were excised, cut into small pieces, subjected to trypsin digestion, and processed for mass spectrometry. Analysis of the APOA4-interacting proteins by mass spectrometry revealed several enriched proteins in the adipose tissue as shown in the volcano and scatter plots (Fig. 2B). While LRP1 was enriched in APOA4 IP vs. IgG controls, it was not the most abundant protein. However, LRP1 was one of the few proteins overall and in the receptor or receptor-like category for which no peptide coverage was detected in IgG vs. APOA4 pull down. Spectral analysis using high-energy collision dissociation–tandem mass spectra confirmed presence of LRP1 in tryptic digests of immunoaffinity pull-downs of anti-APOA4 corresponding to peptides spanning residues L66 to K79 and Y2001 to R2012 of APOA4 and LRP1, respectively (Fig. 2C).

### APOA4-LRP1 interactome

The only known interaction in the STRING database^62^ between APOA4 and LRP1 is derived from the KEGG^63^ pathway for cholesterol metabolism (KEGG: mmu04979), and the Reactome^64^ pathway for retinoid metabolism and transport (R-HSA-975634), which highlight LRP1’s role in membrane transport, but not as a receptor. The interactome for the proteins enriched in the APOA4 IP vs. IgG control IP from our data showed significant interaction between proteins involved in important cellular processes and components, including the ribosome, splicesosome, cytoskeleton, endoplasmic reticulum, and the mitochondrion (Supplementary Fig. S2 online). While previously curated data suggested association between APOA4 and LRP1; no known direct interaction had been shown. Interestingly, our pull down under fasted conditions identified several novel highly enriched proteins that were not reported previously as interacting partners for APOA4 in known databases, such as STRING or KEGG, suggesting that there might be interactions that only occur under certain conditions or in response to specific stimuli (Supplementary Fig. S2 online). When we analyzed proteins enriched with log2 ratio > 1.5 as shown in Fig. 2B, this closer neighborhood around APOA4 confirmed presence of several previously reported proteins that associated with APOA4 complex (Fig. 2D); some of these proteins were moderately enriched in our co-immunoprecipitation pull-down. Based on protein functions, several of these were the expected components of the blood microparticle, cholesterol metabolism, and platelet activation pathways (Fig. 2D). Proteins such as Lipin1 (LPIN1), Talins (TLN1, TLN2), and proline-serine-threonine phosphatase interacting protein 2 (PSTPIP2), which are involved in cytoskeleton organization and components of the peroxisome such as hydroxysteroid dehydrogenase-like 2 (HSDL2), malonyl-CoA decarboxylase (MLYCD), and glyceronephosphate O-acyltransferase (GNPAT) were also enriched, suggesting dynamic context- and function-specific associations.

### Enhanced interaction of APOA4 and LRP1 after lipid feeding in gonadal adipose tissue

**Figure 3:**
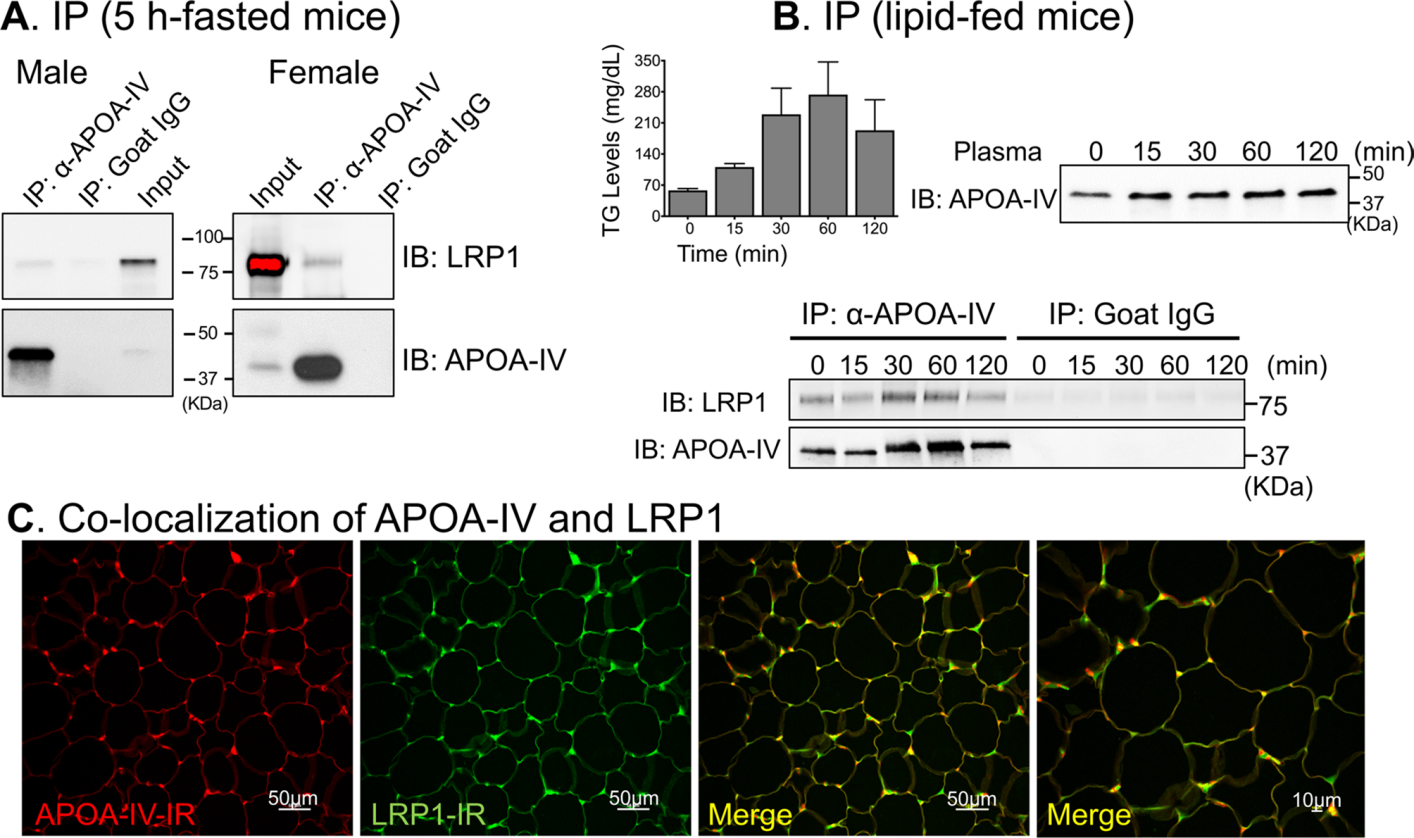
APOA4 and LRP1 interaction increased concomitantly with lipid feeding. (**A**) APOA4 interacted with LRP1 in male (left) and female (right) WT gonadal fat tissues collected 5-h after fasting, as revealed in co-immunoprecipitation assay; pull down with non-specific IgG did not detect LRP1 or APOA4. Representative gel lanes are shown (n = 3). (**B**) Overnight-fasted male mice were gavaged with a single dose of lipid. Epididymal fat tissues were collected before and at 15, 30, 60, 120 min after lipid-gavage and used for co-immunoprecipitation. Triglyceride (TG) levels increased at 15 min by 20% and peaked at 60 min by 44% after lipid gavage, compared to fasted state (0 min). Plasma APOA4 levels also increased by 1.8-fold at 15 min and remained elevated even at 120 min post lipid feeding. APOA4 interaction with LRP1 was enhanced at 30 and 60 min when plasma TG and APOA4 levels were also increased; but no interaction was observed with IgG pull-downs (n = 3). After transfer of proteins, the membranes were cut into two or three sections before probing with specific antibodies for western blots shown. The top section (> 75kD) was used for LRP1 detection, the bottom sections (25–75 kD) for APOA4 detection. This was necessary as stripping and re-probing compromises quantification and running separate gels would not allow for quantification. (**C**) Representative immunofluorescence confocal images of APOA4- and LRP1-immunoreactivity (IR) in epididymal fat tissue sections prepared from male WT mice fasted for 5 h. Robust co-localization (Merge, yellow) of LRP1 (Green) with ApoA4 (Red) was observed in epididymal fat tissue (n = 2 technical and 3 biological replicates). Scale bars: 50 μm and 10 µm.

Among receptor-like proteins identified in our MS analysis, we focused on LRP1 as the receptor for APOA4 for two main reasons: (i) LRP1 is a cell surface and endocytic receptor with robust adipocytes expression, and (ii) adipocyte-specific LRP1 is involved in the regulation of postprandial lipid transport and glucose homeostasis^39^, known functions of APOA4. Next, to confirm our MS findings of LRP1 as an endogenous APOA4-interacting protein, we performed co-IP experiments using either epididymal or periovarian fat tissues of 5-h fasted WT male and female mice, respectively. Western blot analysis showed the presence of both LRP1 and APOA4 bands in α-APOA4, but not IgG pull downs, confirming the interaction (Fig. 3A). Because the interaction between APOA4 and LRP1 was observed in both male and female mice (Fig. 3A), we only used male mice in the subsequent experiments.

Gastric lipid feeding is known to increase plasma APOA4 levels within 30 min^12^. Next, we showed that gavaging male WT mice with a bolus of lipid, caused rapid increases in plasma triglyceride (TG) levels from 58.2 ± 4.1 mg/dL at fasted state (0 min) to 110.7 ± 7.8 mg/dL at 15 min, climbed to 229.3 ± 58.8 mg/dL at 30 min, and reached its maximum levels of 273.7 ± 73.4 mg/dL at 60 min, thereafter fell down to 193.43 ± 68.5 mg/dL at 120 min (Fig. 3B). Western blot of epididymal adipose tissue lysates from 0, 15, 30, 60, 120 min post lipid-feeding revealed that plasma APOA4 levels increased 1.8-fold at 15 min and remained at similar levels until 120 min (Fig. 3B). Immunoprecipitation and immunoblotting confirmed pull down of LRP1 in these lipid-fed mice at all time points, with significant increases in protein levels of both LRP1 and APOA4 at 30 and 60 min, whereas no APOA4 or LRP1 were present in pull down performed using goat IgG (Fig. 3B). These data suggest that lipid feeding results in an increase in protein abundance of APOA4 and an enhancement of APOA4-LRP1 interaction at 30 and 60 min in adipocytes. Immunofluorescence and confocal microscopy further confirmed the co-localization of APOA4 and LRP1 (Fig. 3C) with an overlap coefficient of 0.829 ± 0.02 or ~ 80% colocalization on the cell surfaces of the adipocytes.

### LRP1 mediates glucose uptake by APOA4 in adipocytes

**Figure 4:**
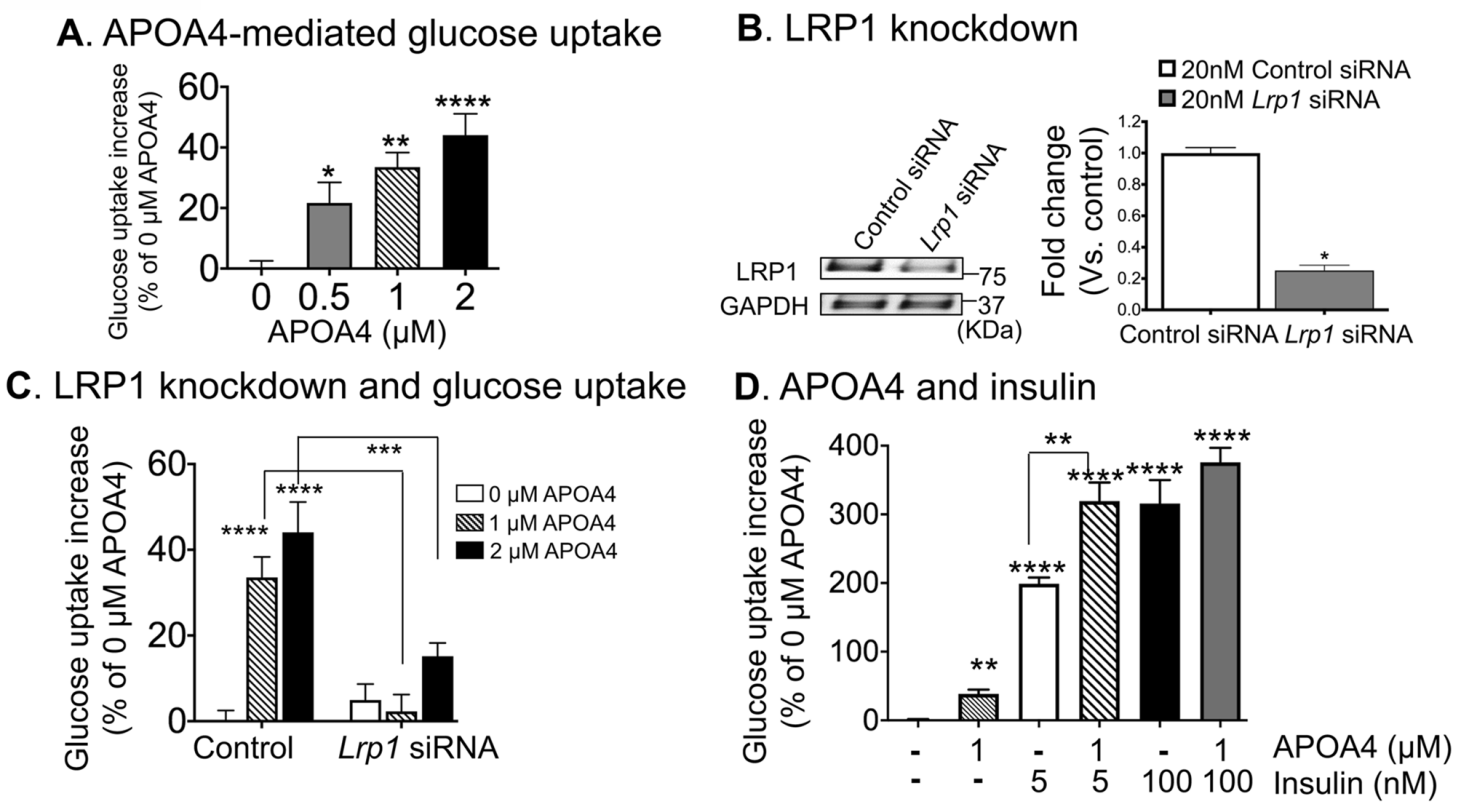
APOA4-stimulated glucose uptake was mediated by LRP1 in 3T3-L1 mature adipocytes. (**A**) APOA4 stimulated glucose uptake by 22–44% in a dose-dependent manner. 3T3-L1 mature adipocytes were incubated with 0, 1, and 2 μM r-m-APOA4 for 1 h (n = 5 per treatment). (**B**) SiRNA targeting *Lrp1* gene was transfected into 3T3-L1 mature adipocytes. Left, a representative western blot image showing reduced LRP1 levels in *Lrp1* siRNA-treated cells, compared to control siRNA-treated cells. GAPDH was used as internal control. Right, quantification of the band intensity revealed ~ 75% decrease in average band intensity for LRP1 protein in *Lrp1* siRNA compared to control siRNA. *p < 0.05, Student’s *t*-test. (**C**) *Lrp1* knockdown abrogated 1 µM and 2 µM APOA4-stimulated glucose uptake by ~ 93% and 67%, respectively, compared with 1 and 2 µM controls. Data are presented as percentage increase of basal glucose uptake levels under control siRNA treatment. **(D)** Insulin-stimulated glucose uptake increased in adipocytes at 5 nM and 100 nM versus untreated. APOA4 (1 µM) treatment further augmented insulin-stimulated glucose uptake at 5 nM, suggesting they act synergistically to promote glucose uptake. Two-way analysis of variance (ANOVA) revealed significant main effects of Apo-A-IV concentration (*p* < 0.0001) and siRNA treatment (*p* < 0.0001) as well as interaction between treatments (p = 0.0007). Sidak’s multiple comparisons test revealed statistically significant changes. ****p < 0.0001, 0 µM vs. 1 and 2 µM (Control), and ***p < 0.001, 1 µM and 2 µM control vs. 1 µM and 2 µM *Lrp1* siRNA, respectively (n = 4–5/treatment).

APOA4 stimulates glucose uptake in an insulin-independent manner in 3T3-L1 mature adipocytes^21^, however, the receptor that facilitates this uptake is not known. To examine if LRP1 is the receptor that mediates APOA4-stimulated glucose uptake, we treated 3T3-L1 adipocytes with r-m-APOA4 and measured glucose uptake rates in both control and *Lrp1* knockdown cells. Treatment of mature adipocytes with increasing concentration (0, 0.5, 1, 2 μM) of APOA4 for 1 h resulted in dose-dependent increase in glucose uptake; ~ 22% increase at 0.5 μM (p < 0.05), ~ 34% at 1 μM (p < 0.01), and ~ 44% at 2 μM (p < 0.0001) of APOA4 compared with buffer (0 μM) treatment (Fig. 4A). Next, transfection of *Lrp1* small interfering RNA (siRNA) resulted in ~ 75% knock-down of LRP1 protein compared to control siRNA (Fig. 4B). *Lrp1* knockdown abrogated APOA4-stimulated glucose uptake by ~ 93% at 1 µM and by ~ 67% at 2 µM (Fig. 4C). These results demonstrated that LRP1 as a cognate receptor for APOA4 that mediates the action of APOA4-stimulated glucose uptake in mature adipocytes. Taken together, these data suggest that dysregulation of LRP1 in peripheral tissues may inhibit peripheral glucose uptake by adipose tissue and possibly by other peripheral organs and may prevent beneficial effects of APOA4 on glucose homeostasis. Reduction of peripheral glucose uptake is one major cause that increases blood glucose levels, thereby contributing to the pathology of obesity, diabetes, and metabolic syndrome.

### APOA4 augments insulin-mediated glucose uptake at low concentrations

Next, we assessed the role of APOA4 in glucose uptake in the presence of graded-doses of insulin. We used two concentrations of insulin: a low dose (5 nM) of insulin mimicking plasma levels seen at postprandial phase^65^ and a high dose (100 nM) used by others to study glucose uptake in 3T3-L1 adipocytes^66^. In mature 3T3-L1 adipocytes, as before, 1 µM APOA4 caused ~ 39% increase in glucose uptake, whereas insulin alone treatment caused ~ 200% and ~ 316% increase in glucose uptake at low (5 nM) and high (100 nM) concentrations, respectively (Fig. 4D). APOA4 plus low dose insulin treatment of adipocytes caused a synergistic effect on glucose uptake: increases to ~ 320% as compared to 39% (APOA4 alone) and 200% (5 nM insulin alone), however at high concentrations of insulin, APOA4 and insulin together did not further augment glucose uptake (Fig. 4D).

### LRP1 modulates APOA4-induced PI3K-AKT phosphorylation

**Figure 5:**
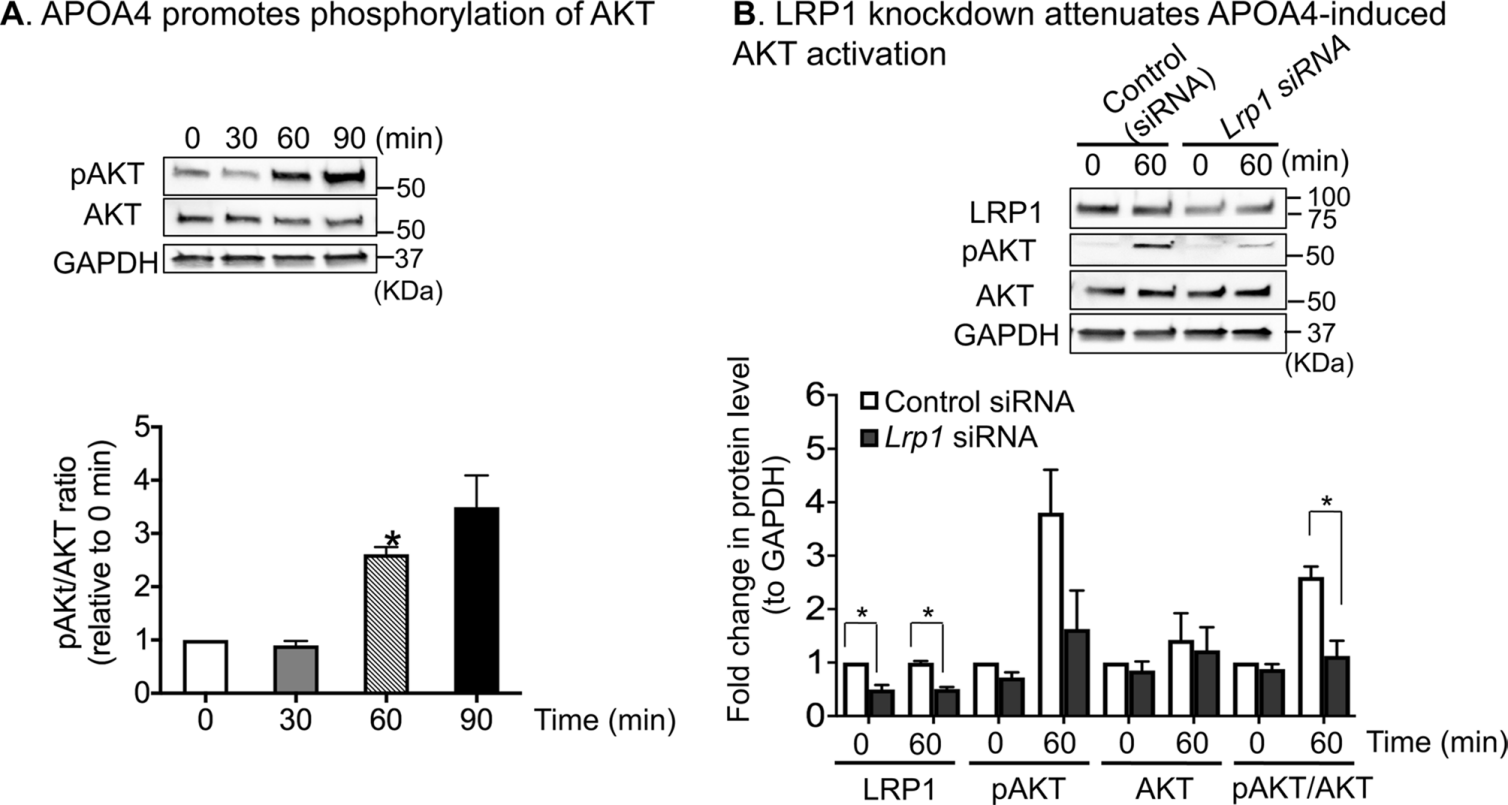
APOA4 activation of PI3K-AKT pathway is partly LRP1-dependent. (**A**) APOA4 promoted phosphorylation of AKT (pAKT) in a time-dependent manner. Representative western blot images are shown. After transfer of proteins, the membranes were cut into three sections before probing with specific antibodies for western blots. The top section (> 75kD) was used for LRP1 detection, the middle sections (50–75 kD) for pAKT and total AKT detection, and the bottom sections (25–50 kD) for GAPDH detection. This was necessary as stripping and re-probing compromises quantification and running separate gels would not allow for quantification. Bar graphs depicting normalized pAKT and AKT levels to GAPDH. Compared to 0 min, the pAKT/AKT ratio was significantly increased by 2.6-fold at 60 min. (**B**) Representative western blot images showing LRP1, pAKT, AKT, and GAPDH. After pAKT detection, the blot was stripped and used for detecting total AKT. At 60 min post APOA4 treatment, when LRP1 levels were reduced by ~ 50% compared with controls, the pAKT/AKT ratio decreased by ~ 58%, suggesting that APOA4-stimulated increases in pAKT are in part, LRP1-dependent (n = 3/group).

Previously we have shown that APOA4 activated PI3K-AKT signaling pathway in 3T3-L1 adipocytes^21^. In this study, we first confirmed that APOA4 treatment of 3T3-L1 adipocytes induced significant increases (~ 2.6-fold) in phosphorylation of AKT (pAKT) at Ser473 site at 60 min (Fig. 5A). In the absence of APOA4 treatment, ~ 18% decrease in pAKT and ~ 10% decrease in total AKT protein levels was observed in adipocytes with LRP1 knockdown compared with control cells, but the difference was not statistically significant. In 3T3-L1 adipocytes with LRP1 knockdown (Fig. 5B), APOA4 treatment caused ~ 1.6-fold increase in pAKT levels, whereas in control adipocytes, APOA4 treatment resulted in a 3.8-fold increase in pAKT levels (Fig. 5B). Similarly, LRP1 knockdown abrogated APOA4-induced increases in pAKT/AKT ratio from 2.6-fold in controls to 1.1-fold in *Lrp1* siRNA-treated adipocytes. Together, the data suggest that the stimulating effect of APOA4 on AKT signaling is, at least partially dependent on LRP1 in 3T3-L1 adipocytes.

## Discussion

Abdominal obesity, dyslipidemia and hyperglycemia are major characteristics of metabolic syndrome, which is known to increase the risk of diabetes and cardiovascular diseases. Patients with type 2 diabetes and metabolic syndrome are at increased risk of SARS-CoV-2 infection and significantly worsened COVID-19 disease outcomes^67^. As a critical player in lipid absorption and metabolism, APOA4 exhibits anti-diabetic and anti-atherogenic effects, rendering it a potential therapeutic target for the treatment of metabolic syndrome^14^ and potentially mitigating the risk of increased SARS-CoV-2 infections in subsets of patients. Given the epidemic proportions of metabolic syndrome and the notion that mitigating comorbidities that might increase one’s risk for worse outcomes, the need to better understand how APOA4 acts on different tissues at the molecular level is paramount. Increased adipose mass contributes to worse outcomes in many clinical settings, including metabolic syndrome; it does so by altering peripheral glucose uptake thereby contributing to insulin resistance, secreting several hormones, altering lipid metabolism and associated signaling^3^. Considering that the majority of APOA4 is secreted from the gut and transported via the blood to various target organs, including adipose tissues, we posit presence of cell/tissue-specific receptor for circulating APOA4 to participate in biological processes at the target tissue. Here, we showed that APOA4 derived from the circulation was taken up by gonadal fat tissue and localized on the cell surfaces of adipocytes. We identified LRP1 as the receptor for APOA4 in both male and female WT 129X1/SvJ mice and the two proteins exhibited robust co-localization in the cell membrane of adipocytes. We demonstrated that APOA4 activated AKT signaling and augmented insulin-independent glucose uptake via LRP1 in 3T3-L1 mature adipocytes; APOA4 together with insulin caused a synergistic increase in glucose uptake. LRP1 knockdown nearly abrogated all APOA4-stimulated glucose uptake and phosphorylation of AKT.

In the present study, we documented that *ApoA4* is not actively transcribed in several tissues; no mRNA for *ApoA4* was amplifiable in the gonadal adipose or kidney tissues of male and female mice by qRT-PCR, whereas abundant mRNA transcripts were amplifiable from jejunum, a key part of the small intestine involved in secretion. Western blot analysis confirmed presence of nearly equivalent levels of APOA4 protein in all tissues examined, including the jejunum, liver, adipose and the kidney. High levels of APOA4 protein were also detected in the plasma, suggesting that tissues, such as the kidney and the gonadal adipose, take up APOA4 from circulation. In agreement with this notion, we also found the presence of APOA4 immunoreactivity in blood vessels and capillaries innervating the subcutaneous, mesenteric, and gonadal fat tissues, along with cell surface expression; no caveolin-1 was present in the blood vessels. It is widely accepted that adipose tissue is not a passive lipid storage organ and adipose tissue dysfunction in the form of excessive lipid deposition is one important underlying cause that contributes to the development of obesity and insulin resistance^3^. When nutrient intake exceeds energetic expenditure for a prolonged period, such as chronic consumption of high-fat diet, excessive energy is stored in adipose tissues, engendering adipocyte hypertrophy and hyperplasia. As adipocytes are enlarged and overloaded with lipids, intracellular abnormalities of adipocyte function occur, particularly endoplasmic reticulum and mitochondria stress^68^. Adipocyte dysfunction results in adipocyte insulin resistance and secretion of non-esterified fatty acids and inflammatory adipokines to the circulation, which trigger fat deposition, lipotoxicity and insulin resistance in remote organs, such as liver, muscle, heart, and vascular beds. In our western blot analysis, adipose APOA4 levels were comparable to that in the plasma whereas total protein loaded onto the gel was ~ 4 times more for adipose compared to plasma, suggesting adipose APOA4 levels were approximately 25% of plasma APOA4 levels. Our finding that a significant amount of APOA4 was targeted to adipose tissue indicated possible influence of APOA4 on adipose function. In fact, the possibility was supported by several lines of evidence. *ApoA4* knockout mice on C57BL/6 J background display reduced uptake of radiolabeled triglyceride-derived fatty acids in epididymal fat tissue compared to WT, suggesting the role of APOA4 in promoting fatty acid uptake in gonadal adipose tissue^69^. Human APOA4 and L-α-dimyristoylphosphatidylcholine complexes promoted cholesterol efflux from cultured adipose cells preloaded with low-density lipoprotein cholesterol^55^. Additionally, our group previously showed that recombinant APOA4 treatment of C57BL/6J mice increased glucose uptake in epididymal and brown adipose tissues^21^. Taken together, these data suggest that APOA4 acts at gonadal fat pad, along with that found in subcutaneous and mesenteric fat pads, to regulate lipid and glucose metabolisms.

After 4 decades since the discovery of APOA4^70–72^, here we report the identification of its first cognate receptor in vivo in adipose tissue. We present unequivocal evidence that APOA4 interacted with LRP1 in vivo using co-immunoprecipitation and combined with mass spectrometry analysis. While LRP1 was not the most abundant protein that was enriched with APOA4, it was the only protein in the “receptor” category that was not present in the IgG pulldowns, but unique spectral counts were identified in APOA4 pulldowns. After identification of LRP1 as a candidate receptor for APOA4 in large-scale mass spectrometry analysis, we confirmed APOA4 interaction with LRP1 using antibodies specifically recognized APOA4 and LRP1 β-subunit. Upon lipid feeding, plasma APOA4 levels increased, and so did the interaction between APOA4 and LRP1. This interaction peaked at 30- and 60-min post lipid-feeding and fell thereafter, which coincided with the timing of highest increases observed in triglyceride levels. This transient interaction pattern between APOA4 and LRP1 is typical of ligand-receptor interaction exhibited by several classes of recognized receptors, including other gut peptides and their receptors^73^.

Our data further implies that presence of lipids may assist in this dynamic interaction between APOA4 and LRP1. It remains to be determined whether free or lipid-bound APOA4, or both forms directly interact with LRP1. It is possible that other apolipoprotein in the chylomicrons^72^, such as APOE, a known LRP1 ligand^74^, facilitates APOA4 interaction with LRP1 during postprandial state. Future studies could include studying the interaction of APOA4 and LRP1 in *ApoE* deficient mice to rule out and/or demonstrate the involvement of APOE in APOA4-LRP1 interactome. Studies might also investigate interaction of APOA4 under different feeding conditions and/or in other organs involved in metabolic processes, such as the liver. In our study, we have identified several novel and previously unreported interacting partners for APOA4 in the adipose tissues (Fig. 2 and Supplementary Fig. S2), but this interactome is likely to be dynamic and is likely to change depending upon feeding state, tissue, and time of interrogation. Immunofluorescence images revealed spatio-temporal co-localization of APOA4 with LRP1, which was at best displayed 80% co-localization coefficient. This is expected and could be attributable to the ability of LRP1 to bind at least 30 different ligands^34^. It is likely that LRP1 interaction with other ligands blocks its interaction with APOA4 under normal or fasted conditions, and thus showing partial co-localization with APOA4, whereas co-localization might increase in a time-dependent manner upon lipid feeding.

Our study showed that APOA4 and LRP1 function in concert to promote insulin-independent glucose uptake via PI3K-AKT pathway in mature adipocytes, establishing the importance of the APOA4-LRP1 axis in regulating glucose metabolism in the absence of insulin. *Lrp1* siRNA treatment led to ~ 50%-75% reduction in LRP1 protein levels compared to control siRNA treatment. Although the knockdown efficiency was not 100%, it was sufficient to abrogate > 90% of glucose uptake at 1 µM and > 60% at 2 µM APOA4, indicating that LRP1 is the cognate receptor for APOA4-mediated glucose uptake. At higher concentrations (2 µM), APOA4 could interact with remaining small levels of (~ 25%) LRP1 proteins in *Lrp1* knockdown cells, allowing for residual glucose uptake. Further elucidation of signaling events downstream of APOA4-LRP1 that facilitate glucose uptake is required to fully understand the role of this novel ligand-receptor complex. We find that in 3T3-L1 cells with LRP1 knockdown, APOA4-stimulated increases in pAKT/AKT ratio were suppressed by ~ 58%, indicating LRP1 is one mediator for APOA4 in activating AKT signaling in adipocytes. Our group previously showed that APOA4 treatment in 3T3-L1 mature adipocytes increased pAKT levels, leading to increased trafficking of glucose transporter type 4 (GLUT4) from intracellular storage vesicles to the plasma membrane^21^. This elevated GLUT4 expression on the cell surface of adipocytes accelerated the binding and uptake of exogenous glucose.

Our data further suggest that APOA4 works synergistically with physiological concentrations of insulin to promote glucose uptake in cultured adipocytes. At a low concentration (5 nM) of insulin, which mimics the postprandial plasma insulin levels, addition of APOA4 increased insulin-stimulated glucose uptake by ~ 60%, whereas no further increases in glucose uptake were seen at a high dose (100 nM) of insulin. This may be attributed to a ceiling effect and to prevent pathological decreases in plasma glucose levels, i.e., hypoglycemia, which has more dire consequences on health. The synergistic interaction between APOA4 and insulin in glucose homeostasis is not unprecedent; we previously reported that APOA4 in conjunction with insulin was able to maintain low blood glucose levels for prolonged periods compared to either APOA4 or insulin alone in WT and KKA^y^ diabetic mice^21^. It is well documented that insulin promotes phosphorylation of insulin receptor substrate family (IRS1-4)^75^. Phosphorylated IRS then interacts with and activates PI3K signaling pathway, which leads to the activation of AKT signaling and eventually GLUT4 translocation^75^. Insulin is known to promote LRP1 and GLUT4 sorting to the plasma membrane via phosphotyrosine interacting domain-containing protein 1 (PID1), a cytosolic adaptor protein of LRP1^76^. We speculate that while insulin promotes glucose uptake through insulin receptor and downstream signaling, insulin also increases the cell surface abundance of LRP1, making them more accessible for interaction with APOA4 to further enhance glucose uptake. Previous research has found that LRP1 was one of the major components in GLUT4-containing storage vesicles and LRP1 deficiency substantially affected GLUT4 expression, resulting in impaired insulin-stimulated glucose uptake^66^. Future studies can address whether APOA4-LRP1 interaction triggers GLUT4 translocation, which is normally regulated by insulin action. Our data sets the stage to decipher the mechanism along APOA4-LRP1 axis in promoting glucose uptake in peripheral tissues in response to food intake and concomitant increases in blood insulin levels.

In conclusion, our work revealed that LRP1 is the cognate receptor for APOA4 in adipocytes that mediates APOA4-induced glucose uptake via PI3K-AKT signaling pathway. As such, our work provides basis for exploring the converging role of APOA4 and LRP1 in lipid metabolism and glucose homeostasis. Moreover, in view of many overlapping functions shared by APOA4 and LRP1, for example, protection against inflammation and atherosclerosis, APOA4 and LRP1 may coordinate to modulate broader spectrum of biological processes in addition to lipid and glucose metabolism, which merits further investigation.

## Methods

### Animals

Male mice on C57BL/6 and 129/SvJ mixed background were a generous gift from Dr. J. L. Breslow (The Rockefeller University, New York, NY)^77^ and were backcrossed to 129X1/SvJ mice obtained from The Jackson Laboratory (Bar Harbor, ME) for 8 generations. Genomic DNA of the 129X1/SvJ *ApoA4*^*−/−*^ mice were extracted and the absence of *ApoA4* were confirmed by PCR amplification. Male and female mice on 129X1/SvJ background were bred and housed at the University of Cincinnati animal facility, maintained in a 12:12-h light–dark cycle at 22 °C with ad libitum access to chow (Teklad 7912 standard chow diet, Teklad Diets, Madison, WI) and water unless specified. Adult male and female (12–16 weeks old) littermates were used for all experiments. All procedures were approved by the Institutional Animal Care and Use Committee (IACUC) at the University of Cincinnati and were in agreement with NIH and ARRIVE guidelines. Lipid gavage: Male 129X1/SvJ wildtype mice were fasted overnight and gavaged with a bolus of lipid emulsion containing 0.22 mL Intralipid 20% (Sigma Aldrich, St. Louis, MO) and 0.08 mL 0.9% saline. Different groups of mice were euthanized at 15, 30, 60 and 120 min after lipid gavage (n = 3/time point). Epididymal fat tissues were then collected for protein extraction and co-immunoprecipitation.

### Quantitative RT-PCR

Total RNA was isolated from the jejunum, liver, gonadal fat, and kidney tissues of adult male and female 129X1/SvJ wildtype mice (n = 3 per sex) using either RNeasy Mini Kit or Lipid Tissue Mini Kit (QIAGEN, Gaithersburg, MD). Real-time PCR was performed in 20μL reaction volume with 2μL of fivefold diluted cDNA, Taqman primers, Taqman Fast Advanced Master mix using a StepOnePlus™ Real-Time PCR system (Life Technologies, Carlsbad, CA) for 38 cycles^78^. The predesigned Taqman Gene Expression Assay IDs (Thermo Fisher Scientific, Rockford, IL) for mouse *ApoA4* (Mm00431814_m1) and β-actin (Mm02619580_g1) were used. See Supplemental Methods for details.

### Western blot analysis

Total proteins were separated on pre-cast 4–15% SDS-PAGE followed by transfer to PVDF membranes and cut before incubation with antibodies. After incubation with the blocking buffer, the following primary antibodies were used: goat polyclonal α-mouse APOA4 (Thermo Fisher Scientific) at 1:10,000 dilution; rabbit α-LRP1 β-subunit (Abcam, EPR3724) at 1:10,000 dilution, rabbit α-phosphoAKT (Cell Signaling Technologies, Cat#4051) at 1:1000 dilution, rabbit α-AKT (Cell Signaling Technologies, Cat#9272) at 1:1000 dilution, rabbit α- β-actin (Cell Signaling Technologies, Cat#4970) at 1:5000 dilution, rabbit α- GAPDH antibodies (Thermo Fisher Scientific, PA1-987) at 1:1000 dilution. Blots were washed and incubated secondary antibodies conjugated with horseradish peroxidase- rabbit anti-goat IgG or goat anti-rabbit IgG antibodies (Agilent Dako-products, Santa Clara, CA) at 1:10,000 dilution. Blots were developed using enhanced chemiluminescence reagents (BioRad Laboratories Inc). Images were captured by ChemiDoc System and band intensities were quantified using Image Lab software.

### Immunofluorescence and confocal microscopy

Fat tissues from age- and sex-matched WT naïve control and 129X1/SvJ *ApoA4*^*−/−*^ mice i.p. injected with 5 μg/g of r-m-APOA4 (n = 3/sex) were fixed in 10% formalin, pH 7.0 for 48 h. Fixed, paraffin-embedded tissues were sectioned, deparaffinized, rehydrated, blocked, and stained as described in detail in Supplemental Methods. For co-staining of APOA4 and caveolin-1, sections were incubated with goat α-mouse APOA4 (1:500) and α-rabbit polyclonal caveolin-1 (1:500; Thermo Fisher Scientific). For co-staining of APOA4 and LRP1, antigen retrieval using citrate protocol was performed; primary antibodies used were as follows: goat α-mouse APOA4 (1:100) and α-rabbit polyclonal LRP1 (1:100; Abcam, Cambridge, MA). Sections were then washed and incubated with either (i) biotinylated anti-goat IgG or (ii) Cy5-conjugated donkey anti-rabbit IgG followed by subsequent incubations with secondary antibodies as described earlier^73^ (Supplemental Methods). Images were acquired using optical sectioning (× 20 or × 40 magnification) to allow for visualization within a given z-plane using Zeiss Imager M2 microscope with Apotome (Carl Zeiss, Thornwood, NY). Images were captured and simultaneously processed (colored and merged) using the Zeiss (Zen 2011) software. Three sets of images (immunostaining for APOA4 and LRP1) taken from two tissue section per mouse (n = 3) were analyzed for co-localization of APOA4 and LRP1 using JACoP plugin^79^ in the free open access software ImageJ (http://rsbweb.nih.gov/ij/).

### Co-immunoprecipitation

For mass spectrometry analysis, co-immunoprecipitation was performed using gonadal fat tissues from adult male and female 129X1/SvJ WT mice (n = 5–6/sex) fasted for a total of 5 h. Three hours after food withdrawal, a single bolus of recombinant mouse APOA4 (2.5 μg/g body weight) was injected i.p. to each mouse, and 2 h later, mice were euthanized and tissues were collected and pooled. Protein was extracted from fat tissues as described in Supplemental Methods in detail. Goat α-mouse APOA4 and goat IgG isotype control (500 μg each) were conjugated to 500 μL of agarose beads as per Amino Link Plus immobilization kit manual (Thermo Fisher Scientific). Ten milligram tissue lysates were incubated with antibody-containing agarose beads overnight at 4 °C. Beads were then loaded onto the empty column and washed with 25 mL protein lysis buffer. Proteins binding to APOA4 antibodies were eluted using 3 fractions of 500 μL Pierce IgG elution buffer (Thermo Fisher Scientific) and the eluate was immediately neutralized with 1.0 M Tris–HCl buffer, pH 9.0.

For co-immunoprecipitation (co-IP) after lipid gavage, protein was prepared from fat tissues from control and lipid-fed mice and protein concentration was determined as detailed in Supplemental Methods. Twenty micrograms α-APOA4 or goat IgG antibodies were added to 500 μg protein from tissue lysate in parallel co-IP reactions and incubated at 4 °C overnight. Lysate-antibody samples were then incubated with pre-washed protein A/G magnetic beads (Thermo Fisher Scientific) for 2 h and washed three times with Hunt buffer. Protein were eluted using Pierce IgG elution buffer (Thermo Fisher Scientific) and subject to western blot analysis to detect LRP1 and APOA4. Detection of non-specific IgG chains were blocked by incubating the blots with horseradish peroxidase-conjugated IgG fraction monoclonal mouse anti-goat IgG, light chain specific (Jackson Immuno-Research laboratories, West Grove, PA); following antibody incubations, blots were developed using enhanced chemiluminescence (BioRad Laboratories Inc., Hercules, CA).

### Mass spectrometry: reverse-phase LC–MS/MS and data analysis

Proteins bound to beads were eluted using Pierce IgG elution buffer (Thermo Fisher Scientific) and neutralized with 1 M Tris–HCl buffer, pH 9.0, and resolved on a 10% SDS-PAGE. In-gel digestion of proteins with trypsin was performed as described previously^80^. Peptides were analyzed in an Orbitrap Lumos Fusion (Thermo Scientific) in positive ion mode. MS spectra were acquired between 375 and 1500 m/z in information-dependent acquisition mode to automatically switch between MS and MS/MS acquisition. For each MS spectrum, multiply charged ions over the selected threshold (2E4) were selected for MSMS in cycles of 3 s. Precursor ions were fragmented by HCD. Peak lists were generated using PAVA software^81^. The peak lists were searched against the murine subset of the SwissProt database. A randomized version of all entries was concatenated to the database to estimate false discovery rates in the searches. Protein Prospector thresholds used for identification criteria were: minimal protein score of 15, minimal peptide score of 15, maximum expectation value of 0.1 and a minimal discriminant score threshold of 0.0. FDR was limited to 1%. Further details about identification of fragments and criteria used have been described by us elsewhere^82,83^ and in Supplemental Methods.

### Network analysis

To create the known APOA4 interactome, the list of all enriched proteins from the co-immunoprecipitation pull-down that were enriched with a ratio of ApoA4-IP/IgG control-IP of > 1.5 and a generous p-value < 0.11 were used to query the STRING database through the Cytoscape^84^ stringApp^85^ with a cutoff of 0.4. The stringApp functional enrichment tool was used to provide enriched terms. Selected terms were used to involve as many proteins as possible in functional roles, avoiding general terms such as “cytoplasm”.

### 3T3-L1 cell culture and siRNA knockdown

The 3T3-L1 murine fibroblast cells (ATCC, Manassas, VA) were seeded in 24-well (for glucose uptake) or 6-well (for detecting phosphoAKT) plates and cultured in high-glucose (4.5 g/L) DMEM supplemented with 10% heat-inactivated fetal bovine serum (FBS) and 1% penicillin–streptomycin (P/S) solution as described previously^86^. Differentiation to mature adipocytes was induced by incubating the cells in culture medium supplemented with 1 μM dexamethasone, 0.5 mM 3-isobutyl-1-methylxanthine (IBMX), 10 μg/mL insulin for 2 days. The cells were subsequently maintained in high-glucose DMEM containing 10% FBS and 1% P/S solution, and used as mature adipocyte on day 7–10 post-IBMX induction. Mature adipocytes were transfected with 20 nM of 27mer *Lrp1* siRNA duplex (rGrCrArArUrGrUrGrArCrUrArCrGrArCrArArCrGrArCrUGC) or control siRNA duplex (Universal scrambled Cat# SR423695) according to manufacturer’s instruction (ORIGENE, Rockville, MD). Post siRNA transfection, cells were maintained in DMEM containing 10% FBS and 1% P/S for 72–96 h before any r-m APOA4 treatment (provided by Dr. Davidson’s laboratory).

### Glucose uptake assay

Cells were washed three times with PBS and starved in Krebs–Ringer-Phosphate-Hepes (KRPH) with 2% BSA buffer for 40 min. The cells were then treated with 0, 1 and 2 μM recombinant mouse APOA4 (provided by Dr. W. Sean Davidson at University of Cincinnati) for 1 h. Alternatively, cells were treated with either insulin (Sigma, Cat#SLCB9851) or APOA4 or both for 1 h. The glucose uptake rates were measured as the amount of 2-deoxyglucose-6-phosphate in the aliquoted cell lysate using glucose uptake assay kit (Abcam, Cambridge, MA) following manufacturer’s manual. Protein concentration in cell lysates were determined using Pierce 660 nM protein assay kit. Glucose uptake rates were normalized to total protein amount in the cell lysate used for 2-DG6P measurement as detailed in Supplemental Methods. Data was presented as percentage increase of basal glucose uptake rates under control siRNA treatment (n = 5/treatment). Ten microgram total protein from the cell lysate was used for western blot analysis to assess the degree of LRP1 knockdown after *Lrp1* siRNA transfection. The band intensities of LRP1 was normalized to that of GAPDH. Data was presented as fold change over the normalized LRP1 levels in siRNA control-treated cells.

### APOA4 treatment of 3T3-L1 adipocytes for analysis of AKT

Control and *Lrp1* siRNA pre-treated cells were stimulated with 1 μM APOA4 for 1 h. Cells at 0 min and 60-min post-treatment were washed and lysed in buffer (see details in Supplemental Methods). Fifteen microgram total protein were used for western blot analysis to detect LRP1, pAKT and GAPDH. The blot for pAKT detection was stripped and re-probed with antibodies against total AKT as described in the western blot section. Protein bands of interest was normalized to GAPDH. Data was presented as fold change over the normalized protein levels in siRNA control-treated cells at 0 min.

### Statistical analysis

Data are presented as the mean ± SEM. Intergroup differences were compared using a one- or two-way analysis of variance (ANOVA) followed by the Dunnett or Sidak’s test where appropriate. Two group difference was performed using independent t-tests. A p value of less than 0.05 was considered as statistically significant, the level of significance was indicated as *p < 0.05, **p < 0.01, ***p < 0.001, ****p < 0.0001.

## Supplementary Information

Supplementary Information 1.

Supplementary Information 2.

## Data Availability

All relevant data are included in the article and/or its supplementary information file and figures.
