# Supplementary material for: Low-density lipoprotein receptor-related protein 1 (LRP1) is a novel receptor for apolipoprotein A4 (APOA4) in adipose tissue
Source: Sci Rep. 2021 Jun 24;11:13289. doi: 10.1038/s41598-021-92711-0 (PMC8225859; doi:10.1038/s41598-021-92711-0)
Supplement: Supplementary file 1 — Supplementary Information 1. [file 41598_2021_92711_MOESM1_ESM.pptx]

## Slide 1
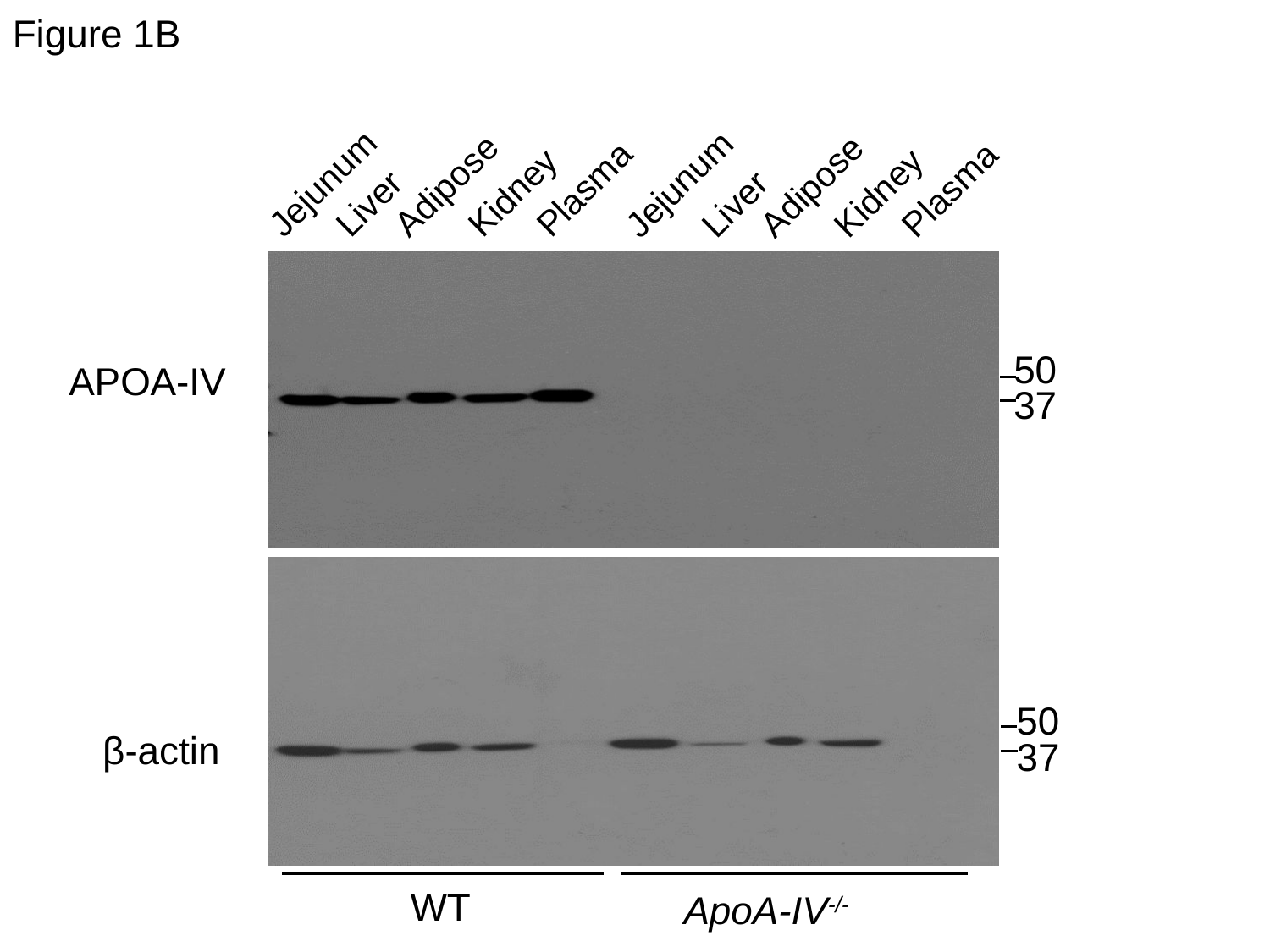

Figure 1B
Jejunum
Kidney
Adipose
Plasma
Liver
Jejunum
Kidney
Adipose
Plasma
Liver
50
APOA-IV
37
50
37
β-actin
WT
ApoA-IV-/-

## Slide 2
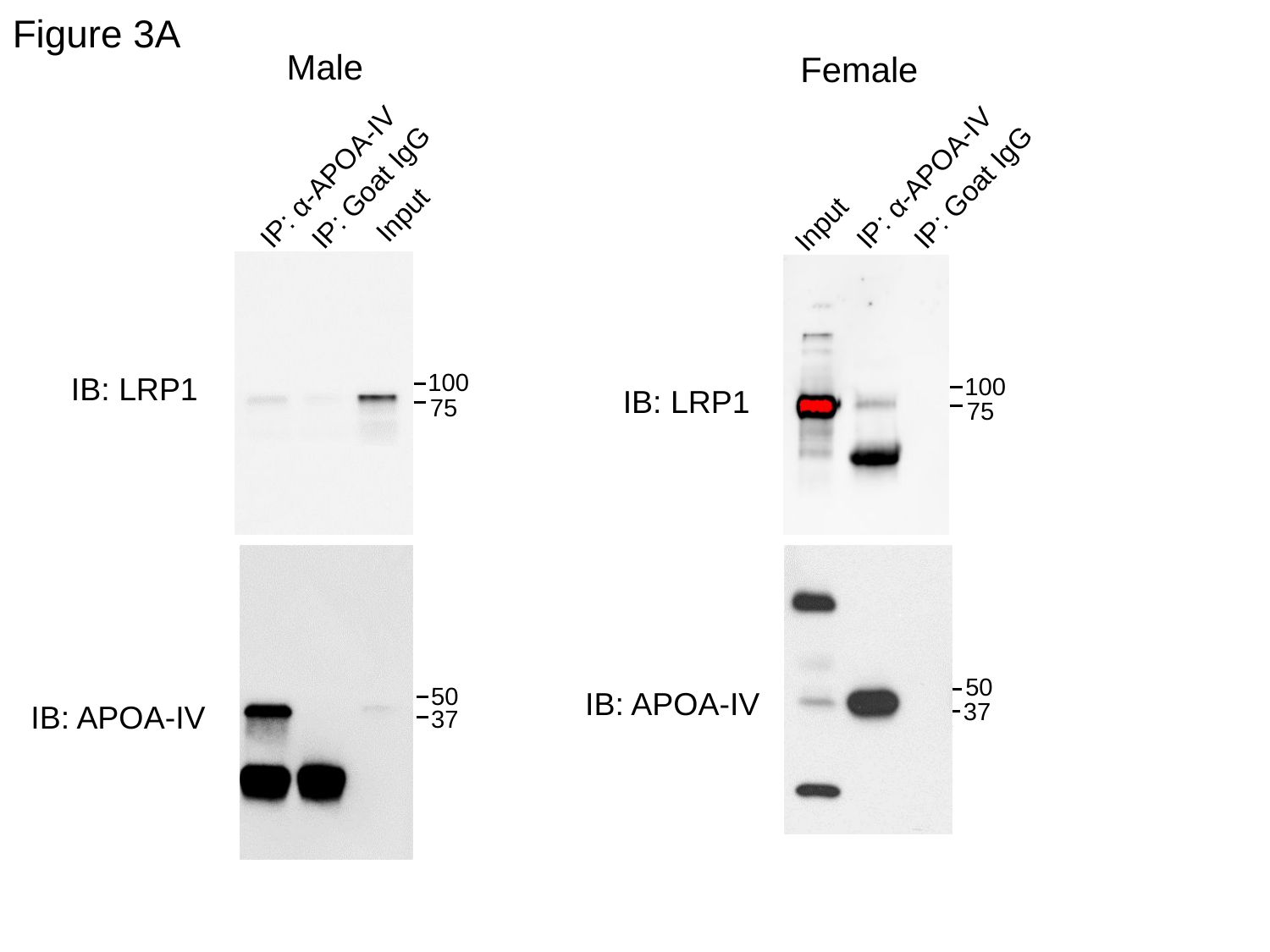

Figure 3A
Male
Female
Input
IP: α-APOA-IV
IP: Goat IgG
IP: α-APOA-IV
IP: Goat IgG
Input
100
IB: LRP1
100
75
IB: LRP1
75
50
50
IB: APOA-IV
37
IB: APOA-IV
37

## Slide 3
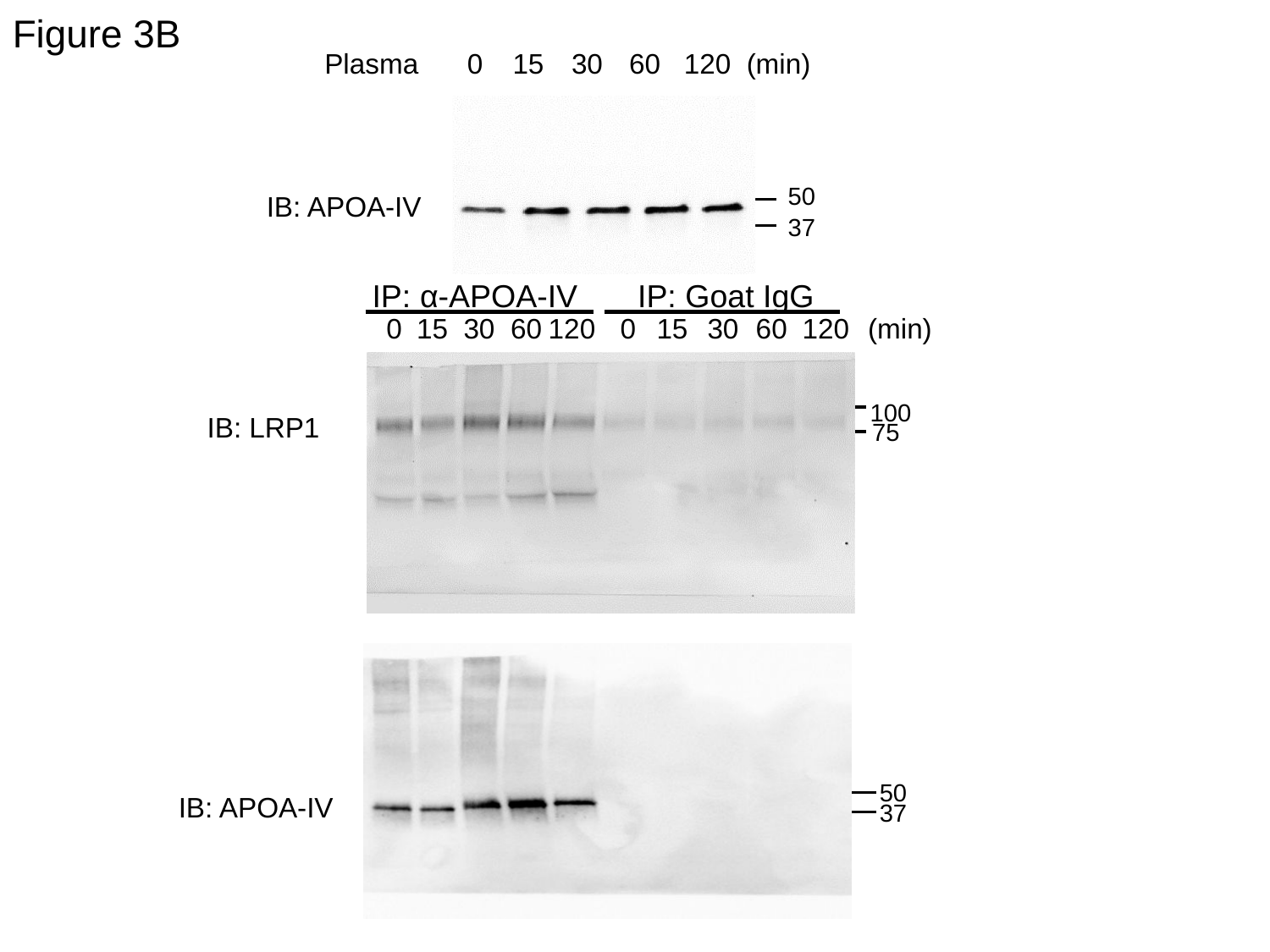

Figure 3B
Plasma
0
15
30
60
120 (min)
50
37
IB: APOA-IV
IP: α-APOA-IV
IP: Goat IgG
0
15
30
60
120
0
15
30
60
120
(min)
IB: LRP1
100
75
50
37
IB: APOA-IV

## Slide 4
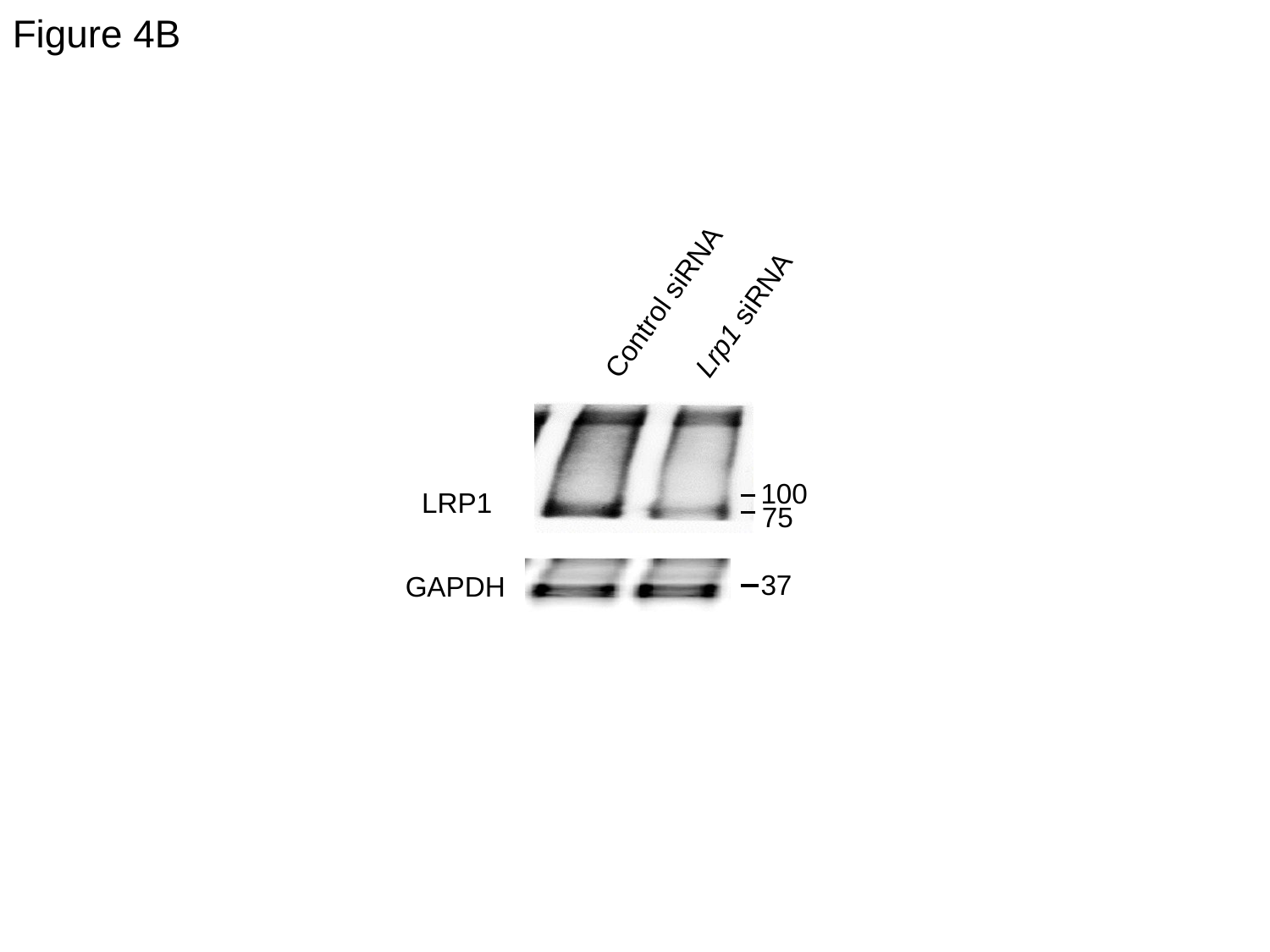

Figure 4B
Control siRNA
Lrp1 siRNA
LRP1
100
75
37
GAPDH

## Slide 5
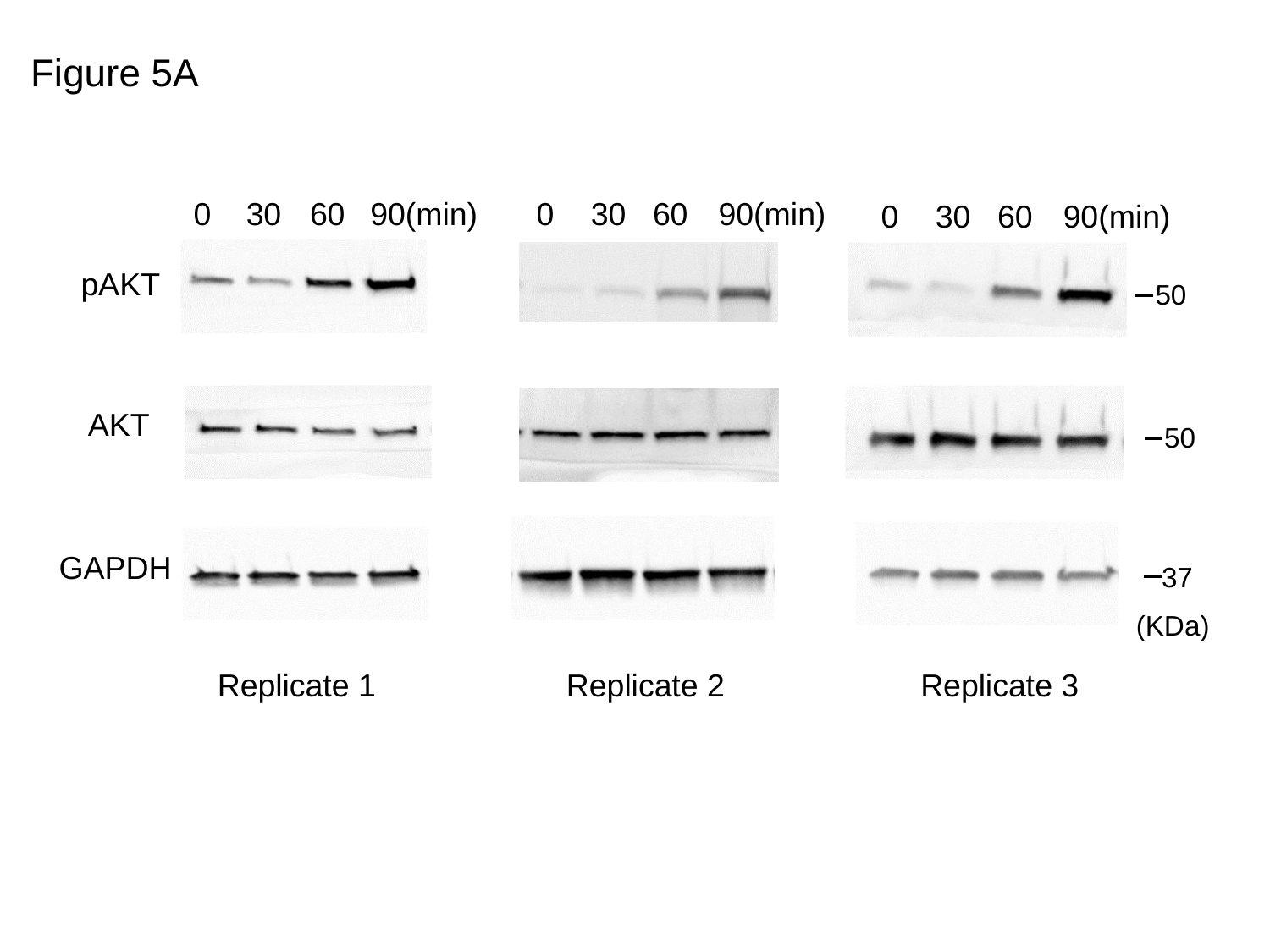

Figure 5A
0
30
60
90(min)
0
30
60
90(min)
0
30
60
90(min)
pAKT
50
AKT
50
GAPDH
37
(KDa)
Replicate 1
Replicate 2
Replicate 3

## Slide 6
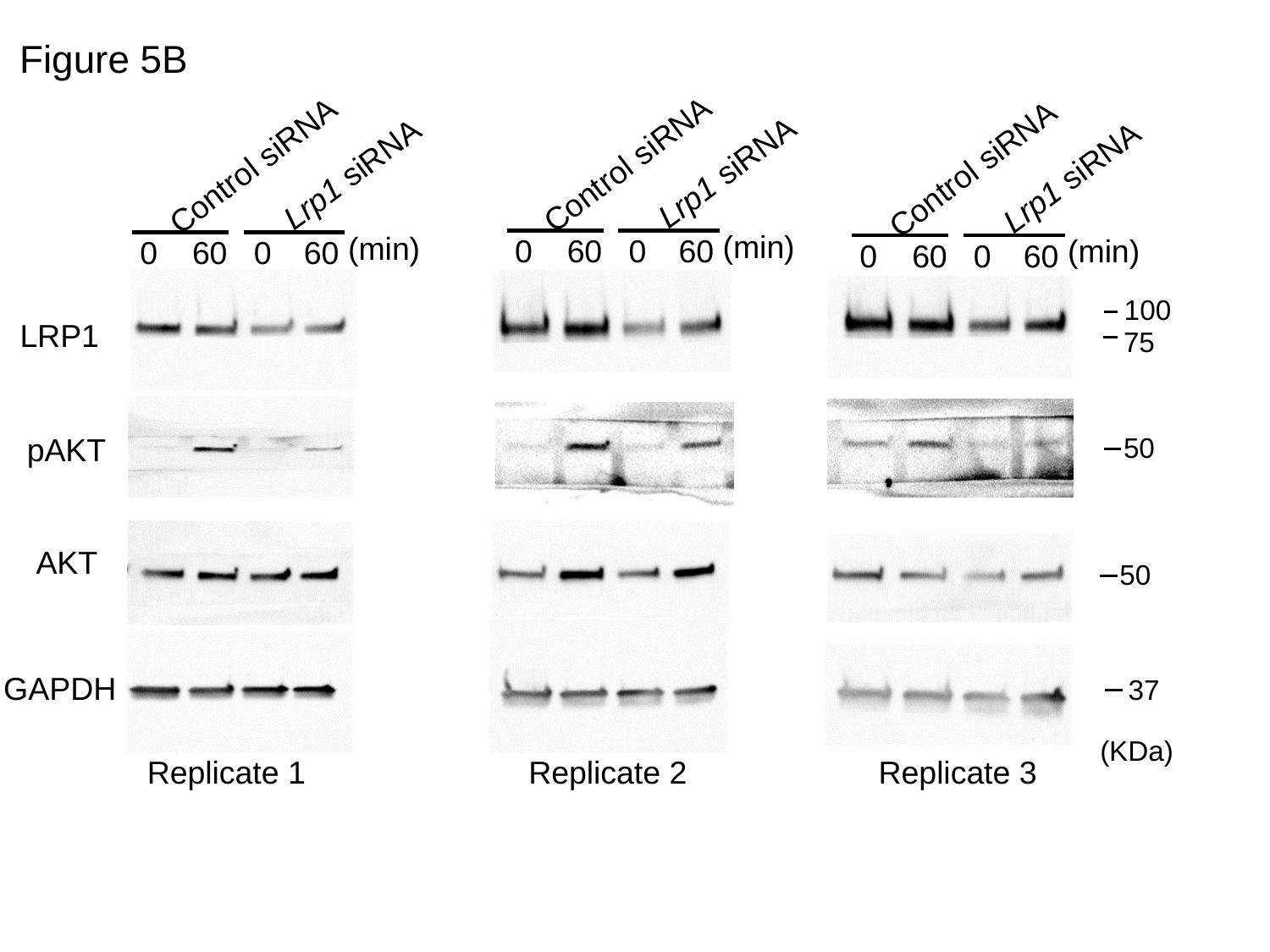

Figure 5B
Control siRNA
Lrp1 siRNA
(min)
0
60
0
60
Control siRNA
Lrp1 siRNA
(min)
0
60
0
60
Control siRNA
Lrp1 siRNA
(min)
0
60
0
60
100
LRP1
75
pAKT
50
AKT
50
GAPDH
37
(KDa)
Replicate 1
Replicate 2
Replicate 3
