# Supplementary material for: Low-density lipoprotein receptor-related protein 1 (LRP1) is a novel receptor for apolipoprotein A4 (APOA4) in adipose tissue
Source: Sci Rep. 2021 Jun 24;11:13289. doi: 10.1038/s41598-021-92711-0 (PMC8225859; doi:10.1038/s41598-021-92711-0)
Supplement: Supplementary file 2 — Supplementary Information 2. [file 41598_2021_92711_MOESM2_ESM.docx]

**Low-density lipoprotein receptor-related protein 1 (LRP1) is a novel receptor for apolipoprotein A-4 (APOA4) in adipose tissue**

**Jie Qu^1^, Sarah Fourman^1^, Maureen Fitzgerald^1^, Min Liu^1^, Supna Nair^2^, Juan Oses-Prieto^2^, Alma Burlingame^2^, John H. Morris^2^, W. Sean Davidson^1^, Patrick Tso^1+^and Aditi Bhargava^3+^***

^1^Department of Pathology and Laboratory Medicine, University of Cincinnati College of Medicine, 2180 E Galbraith Road, Cincinnati 45237-0507, USA.

^2^Departments of Pharmaceutical Chemistry, University of California San Francisco, 600 16th Street, San Francisco, CA 94158, USA.

^3^Department of Obstetrics and Gynecology & Center for Reproductive Sciences, University of California San Francisco, 513 Parnassus Avenue, San Francisco, CA 94143-0556, USA.

**^+^**: Co-senior authors

*: Correspondence:

Aditi Bhargava, PhD

Department of Obstetrics and Gynecology,

Center for Reproductive Sciences,

513 Parnassus Avenue, Rm HSE1636

University of California San Francisco,

San Francisco, CA 94143-0556, USA.

Phone: (415) 502-8453.

Email: aditi.bhargava@ucsf.edu

**Supplemental Materials and Methods**

**Quantitative RT-PCR**

Adult male and female 129X1/SvJ wildtype (WT) mice (n=3 per sex) were fasted for 5 h and then euthanized. Jejunum, liver, gonadal fat tissues, and kidney were collected and rapidly frozen in liquid nitrogen for RNA or protein analyses. Blood was collected for plasma preparation. Total RNA was isolated using either RNeasy Mini Kit or Lipid Tissue Mini Kit (QIAGEN, Gaithersburg, MD), followed by removal of genomic DNA with a DNA-free kit (Ambion, Grand Island, NY). Complementary DNA was prepared from 1μg of total RNA using iScript cDNA synthesis kit (BioRad Laboratories Inc., Hercules, CA), according to the manufacturer’s protocol. Twenty microliter reactions were diluted five-fold and 2 μL of the dilutions were used for subsequent real-time PCR reactions. Real-time PCR was performed in 20 μL reaction volume with cDNA, Taqman primers, Taqman Fast Advanced Master mix using a StepOnePlus Real-Time PCR system (Life Technologies, Carlsbad, CA). The following cycling conditions were used: hot-start at 95°C for 3 min for one cycle, followed by 38 cycles of denaturing at 95°C for 30 sec and annealing/extension at 60°C for 20 sec^1^. The predesigned Taqman Gene Expression Assay IDs (Thermo Fisher Scientific, Rockford, IL) for mouse *ApoA4* (Mm00431814_m1) and β-actin (Mm02619580_g1) were used.

**Western blot analysis**

Total proteins were separated on pre-cast 4-15% SDS-PAGE followed by transfer to PVDF membranes. For western blots shown in Fig.1-3, the edge of the full blots was not visible due to short exposure time to X-ray film or in ChemiDoc System (BioRad Laboratories Inc). After transfer of proteins, the membranes were cut into two or three sections before probing with specific antibodies for western blots shown in Fig. 4 and 5, as indicated. The top section (>75kD) was used for LRP1 detection, the middle section (50-75 kD) for pAKT and total AKT detection, and the bottom section (25-50 kD) for GAPDH detection. This was necessary as stripping and re-probing compromises quantification and running separate gels would not allow for quantification. After incubation with the blocking buffer, the following primary antibodies were used: goat polyclonal α-mouse APOA4 (Thermo Fisher Scientific) at 1:10000 dilution; rabbit α-LRP1 β-subunit (Abcam, EPR3724) at 1:10000 dilution, rabbit α-phosphoAKT (Cell Signaling Technologies, Cat#4051) at 1:1000 dilution, rabbit α-AKT (Cell Signaling Technologies, Cat#9272) at 1:1000 dilution, rabbit α- β-actin (Cell Signaling Technologies, Cat#4970) at 1:5000 dilution, rabbit α- GAPDH antibodies (Thermo Fisher Scientific, PA1-987) at 1:1000 dilution. Blots were washed and incubated secondary antibodies conjugated with horseradish peroxidase- rabbit anti-goat IgG or goat anti-rabbit IgG antibodies (Agilent Dako-products, Santa Clara, CA) at 1:10000 dilution. Blots were developed using enhanced chemiluminescence reagents (BioRad Laboratories Inc). Images were captured by ChemiDoc System and band intensities were quantified using Image Lab software.

**Immunofluorescence and confocal microscopy**

Adult male and female 129X1/SvJ *ApoA-IV^-/-^* mice (n=3/sex) were injected i.p. with a single dose of r-m-APOA4 (5 μg/g body weight) at 2 h before euthanasia, and then tissues were collected as described above. Age- and sex-matched WT mice were used as controls (n=3/sex). Fat tissues were fixed in 10% formalin, pH 7.0 for 48 h at 4°C, followed by soaking in 70% ethanol at 4°C for 24 h. Fixed tissues were paraffin-embedded and sectioned at the Pathology Research Core at Cincinnati Children’s Hospital (Cincinnati, OH). For immunostaining, sections were deparaffinized by incubating the slides at 60°C for 20 min, followed by 5 min incubation in xylene twice. Slides were rehydrated by immersion in 1:1 xylene: ethanol, 100% ethanol twice, 95%, 70%, and 50% ethanol sequentially for 5 min in each solution, with a final rinse in deionized water. Sections were rinsed in phosphate-buffered saline (PBS), incubated in 3% hydrogen peroxide for 15 min, rinsed, and blocked with PBS containing 4% horse serum, 0.2% BSA and 0.4% TritonX-100 for 2 hours. For co-staining of APOA4 and caveolin-1, sections were incubated with goat α-mouse APOA4 (1:500) and α-rabbit polyclonal caveolin-1 (1:500; Thermo Fisher Scientific) overnight at room temperature after blocking for 2 hours. Sections were then washed, incubated with biotinylated anti-goat IgG (1:400; BA-9500; Vector Laboratories, Burlingame, CA) and Cy5-conjugated donkey anti-rabbit IgG (1:400; Jackson Immuno-Research, West Grove, PA) for 1 h, followed by washes and incubation with Cy-3 Streptavidin (1:500; Thermo Fisher Scientific, Waltham, MA) for 45 min. Slides were rinsed in deionized water and coverslipped using a polyvinyl alcohol mounting medium with DABCO (10981; Millipore Sigma). For co-staining of APOA4 and LRP1, antigen retrieval using citrate protocol was performed as described before and sections were processed as above; primary antibodies used were as follows: goat α-mouse APOA4 (1:100) and α-rabbit polyclonal LRP1 (1:100; Abcam, Cambridge, MA) overnight. Next days, sections were washed and incubated with biotinylated horse anti-goat IgG (1:500; BA-9500; Vector Laboratories, Burlingame, CA) and Cy-3 Streptavidin (1:500; Thermo Fisher Scientific) and Alexa Fluor® 488-conjugated goat anti-rabbit IgG (1:1000; Abcam, Cambridge, MA) secondary antibodies as described earlier^2^. For immunohistochemical staining of APOA4 using DAB (3,3′-Diaminobenzidine), after overnight primary antibody incubation, sections were incubated with biotinylated horse anti-goat IgG (1:400; BA-9500; Vector Laboratories, Burlingame, CA) for 1 h, followed by incubating in ABC peroxidase solution (1:800; Cat#32020, Thermo Fisher Scientific) for 1 h and DAB (D5905, Millipore Sigma) solution. Sections were rinsed in PBS several times, rehydrated by immersion in 50%, 95%, 100% ethanol twice, 1:1 xylene:100% ethanol, xylene twice sequentially for 5 min in each solution. Slides were coverslipped using a DPX mounting medium (44581; Millipore Sigma). Images were acquired using optical sectioning (x20 or x40 magnification) to allow for visualization within a given z-plane using Zeiss Imager M2 microscope with Apotome (Carl Zeiss, Thornwood, NY). Images were captured and simultaneously processed (colored and merged) using the Zeiss (Zen 2011) software. Three sets of images (immunostaining for APOA4 and LRP1) taken from two tissue section per mouse (n=3) were analyzed for co-localization of APOA4 and LRP1 using JACoP plugin^3^ in NIH ImageJ (http://rsbweb.nih.gov/ij/).

**Co-immunoprecipitation**

For mass spectrometry analysis, co-immunoprecipitation was performed using gonadal fat tissues from adult male and female 129X1/SvJ WT mice (n=5-6/sex) fasted for a total of 5 h. Three hours after food withdrawal, a single bolus of recombinant mouse APOA4 (2.5 μg/g body weight) was injected i.p. to each mouse, and 2 h later, mice were euthanized and tissues were collected and pooled. Fat tissues were minced on dry ice and suspended in ice-cold protein lysis buffer containing T-PER tissue protein extraction reagent, 5 mM 2-mercaptoethanol, and 1X protease inhibitor cocktail. Tissues were homogenized on ice using IKA T-25 ULTRA-TURRAX digital high-speed homogenizer systems (Sigma Aldrich), followed by centrifugation at 600 g for 2 min at 4°C. Fat layer was removed and the supernatant was further centrifuged at 10,000 g for 30 min at 4°C. After centrifugation, the supernatant was collected and protein concentration was determined using Pierce 660 nM protein assay kit (Thermo Fisher Scientific). Goat α-mouse APOA4 and goat IgG isotype control (500 μg each) were conjugated to 500μL of agarose beads as per the protocol specified in the Amino Link Plus immobilization kit manual (Thermo Fisher Scientific). Ten milligram tissue lysates were incubated with antibody-containing agarose beads overnight at 4°C. Beads were then loaded onto the empty column and washed with 25 mL protein lysis buffer. Proteins binding to APOA4 antibodies were eluted using 3 fractions of 500 μL Pierce IgG elution buffer (Thermo Fisher Scientific) and the eluate was immediately neutralized with 1.0 M Tris-HCl buffer, pH 9.0. The eluates were combined and concentrated using Pierce protein concentrator with 3 kDa cutoff (Thermo Fisher Scientific). One tenth of the concentrated eluates were subjected to SDS-PAGE and silver staining, and the rest of the eluates were used for mass spectrometry analysis.

For co-immunoprecipitation (co-IP) after lipid gavage, fat tissues from control and lipid-fed mice were suspended in HUNT buffer (20 mM Tris–HCl pH 8.0, 100 mM NaCl, 1 mM EDTA, 0.5% NP-40, 50 mM NaF, 1X protease and phosphatase inhibitors) and homogenized using a Dounce homogenizer. After centrifugation at 600 g for 2 min at 4°C, the liquid phase was aspirated and centrifuged at 10,000 g for 30 min at 4°C. The supernatant was collected and protein concentration was determined using Pierce 660 nM protein assay kit (Thermo Fisher Scientific). Twenty micrograms α-APOA4 (rabbit or goat anti-mouse) or anti-goat/rabbit IgG antibodies were added to 500 μg protein from tissue lysate in parallel co-IP reactions and incubated at 4°C overnight. Lysate-antibody samples were then incubated with pre-washed protein A/G magnetic beads (Thermo Fisher Scientific) for 2 h and washed three times with Hunt buffer. Protein were eluted using Pierce IgG elution buffer (Thermo Fisher Scientific) and subject to western blot analysis to detect LRP1 and APOA4. Detection of non-specific IgG chains were blocked by incubating the blots with horseradish peroxidase-conjugated IgG fraction monoclonal mouse anti-goat IgG, light chain specific (Jackson Immuno-Research laboratories, West Grove, PA); following antibody incubations, blots were developed using enhanced chemiluminescence (BioRad Laboratories Inc., Hercules, CA).

**Mass Spectrometry: Reverse-phase LC-MS/MS and Data Analysis**

Proteins bound to beads were eluted using Pierce IgG elution buffer (Thermo Fisher Scientific) and neutralized with 1 M Tris-HCl buffer, pH 9.0, and resolved on a 10% SDS-PAGE. In-gel digestion of proteins with trypsin was performed as described previously^4^. Peptides were analyzed in an Orbitrap Lumos Fusion (Thermo Scientific) in positive ion mode. MS spectra were acquired between 375 and 1500 m/z in information-dependent acquisition mode to automatically switch between MS and MS/MS acquisition. For each MS spectrum, multiply charged ions over the selected threshold (2E4) were selected for MSMS in cycles of 3 seconds. Precursor ions were fragmented by HCD. A dynamic exclusion window was applied which prevented the same m/z from being selected for 1 min after its acquisition. Peak lists were generated using PAVA software^5^. The peak lists were searched against the murine subset of the SwissProt database as of April 8, 2019, using in-house Protein Prospector version 5.2.2 (a public version is available online). A randomized version of all entries was concatenated to the database to estimate false discovery rates in the searches. Peptide tolerance in searches was 20 ppm for precursor ions, and 0.8 Da for product ions, respectively. Missing peptide spectral matches were assigned 0.5 values and APOA4 pull down peptide spectral matches were normalized to control IgG pull down to calculate average log 2 ratio values shown for ApoA4/control. Further details about identification of fragments and criteria used have been described by us elsewhere^6,7^.

**3T3-L1 cell culture and siRNA knockdown**

The 3T3-L1 murine fibroblast cells (ATCC, Manassas, VA) were seeded in 24-well (for glucose uptake) or 6-well (for detecting phosphoAKT) plates and cultured in high-glucose (4.5 g/L) DMEM supplemented with 10% heat-inactivated fetal bovine serum (FBS) and 1% penicillin-streptomycin (P/S) solution as described previously^8^. Differentiation to mature adipocytes was induced by incubating the cells in culture medium supplemented with 1μM dexamethasone, 0.5 mM 3-isobutyl-1-methylxanthine (IBMX), 10 μg/mL insulin for 2 days. The cells were subsequently maintained in high-glucose DMEM containing 10% FBS and 1% P/S solution, and used as mature adipocyte on day 7-10 post-IBMX induction. Mature adipocytes were transfected with 20 nM of 27mer *Lrp1* siRNA duplex (rGrCrArArUrGrUrGrArCrUrArCrGrArCrArArCrGrArCrUGC) or control siRNA duplex (Universal scrambled Cat# SR423695) according to manufacturer’s instruction (ORIGENE, Rockville, MD). Post siRNA transfection, cells were maintained in DMEM containing 10% FBS and 1% P/S for 72-96 h before any APOA4 treatment.

**Glucose uptake assay**

Cells were washed three times with PBS and starved in Krebs-Ringer-Phosphate-Hepes (KRPH) with 2% BSA buffer for 40 mins. The cells were then treated with 0, 1 and 2 μM recombinant mouse APOA4 for 1 h. Alternatively, cells were treated with either insulin (Sigma, Cat#SLCB9851) or APOA4 or both for 1 h. The glucose uptake rates were measured as the amount of 2-deoxyglucose-6-phosphate in the aliquoted cell lysate using glucose uptake assay kit (Abcam, Cambridge, MA) following manufacturer’s manual. Protein concentration in cell lysates were determined using Pierce 660 nM protein assay kit. Glucose uptake rates were normalized to total protein amount in the cell lysate used for 2-DG6P measurement as detailed in Supplemental Methods. Data was presented as percentage increase of basal glucose uptake rates under control siRNA treatment (n=5/treatment). Ten microgram total protein from the cell lysate was used for western blot analysis to assess the degree of LRP1 knockdown after *Lrp1* siRNA transfection. The band intensities of LRP1 was normalized to that of GAPDH. Data was presented as fold change over the normalized LRP1 levels in siRNA control-treated cells.

**APOA4 treatment of 3T3-L1 adipocytes for analysis of AKT**

Control and *Lrp1* siRNA pre-treated cells were stimulated with 1 μM APOA4 for 1 h. Cells at 0 min and 60-min post-treatment were washed in PBS for three times and lysed in buffer containing 20 nM Tris, pH 7.4, 137 mM NaCl, 2 nM EDTA, 10% glycerol, 1% Triton X-100, 1X protease and phosphatase inhibitors. Fifteen microgram total protein were used for western blot analysis to detect LRP1, pAKT and GAPDH. The blot for pAKT detection was stripped and re-probed with antibodies against total AKT using dilutions as described in the western blot section. Briefly, full blots in Fig.5 were cut into three strips, >75 kD for detecting LRP1, 50-75 kD for pAKT and 25-50 kD for GAPDH. After pAKT detection, the blots were stripped and used for detecting total AKT. Protein bands of interest was normalized to GAPDH. Data was presented as fold change over the normalized protein levels in siRNA control-treated cells at 0 min.

**Supplemental Figures**.


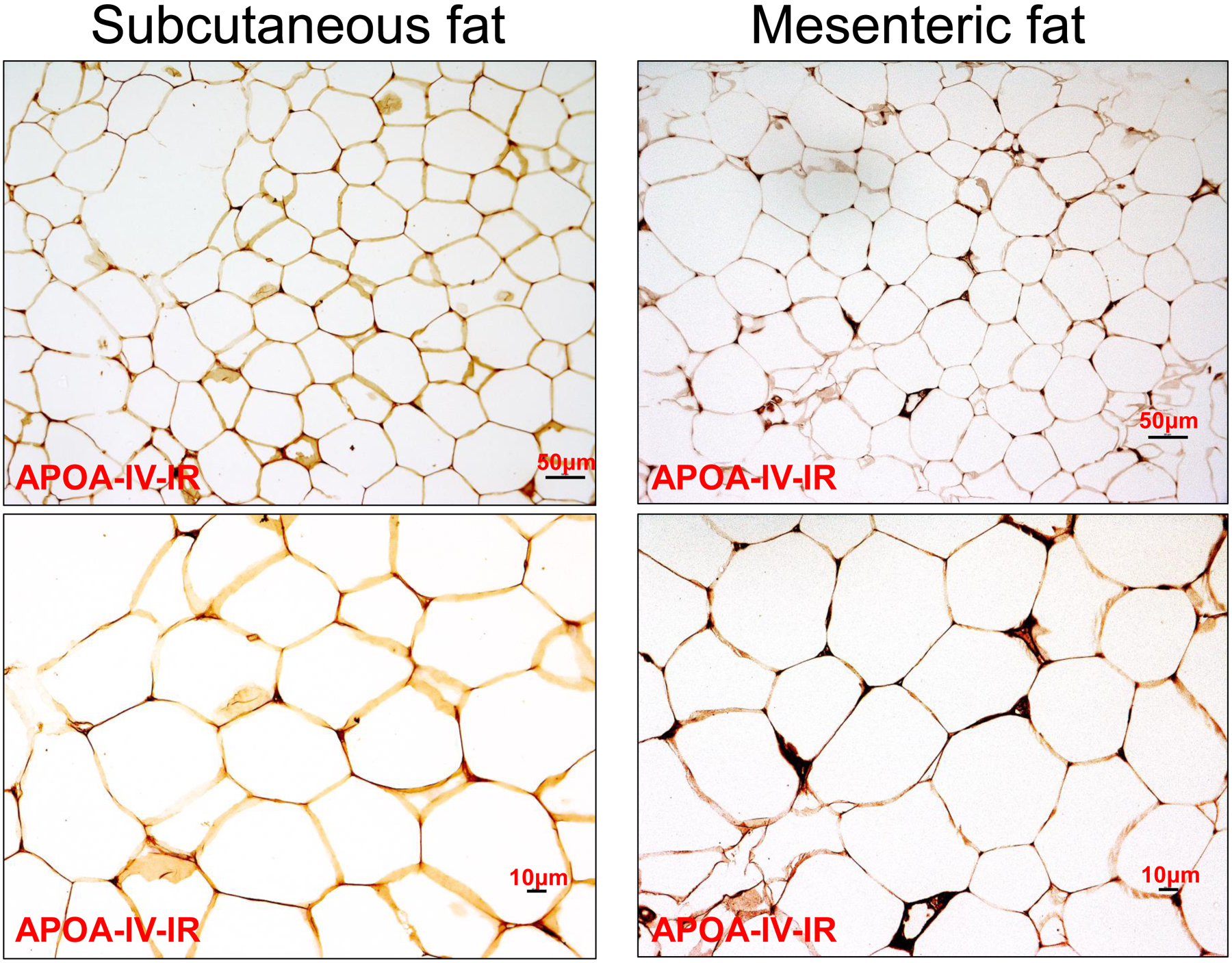


**Supplemental Figure S1 Legend**: Immunohistochemical staining and light microscopy showed that APOA4 immunoreactivity (indicated by dark brown precipitate) was present at the cell surface of adipocytes in a discrete manner in male subcutaneous (Left) and mesenteric (Right) fat tissues. Scale bar: 50μm and 10μm.

**Supplemental Figure S2 Legend**: STRING protein-protein interactions for proteins with APOA4-IP/IgG control-IP ratios > 1.5. Nodes are colored by Log2 (IP/Control) except for APOA4, which is colored grey to stand out. Larger nodes correspond to the highlighted proteins in Figure 2B. Obvious groups of nodes were annotated using STRING GO enrichment of only those nodes, except for the neighborhood around APOA4, which was more fully annotated in Figure 2D.

**Supplemental References**

1 Shen, L. *et al.* Ginsenoside Rb1 increases insulin sensitivity by activating AMP-activated protein kinase in male rats. *Physiol Rep* **3**, doi:10.14814/phy2.12543 (2015).

2 Hasdemir, B., Mahajan, S., Bunnett, N. W., Liao, M. & Bhargava, A. Endothelin-converting enzyme-1 actions determine differential trafficking and signaling of corticotropin-releasing factor receptor 1 at high agonist concentrations. *Mol Endocrinol* **26**, 681-695, doi:10.1210/me.2011-1361 (2012).

3 Bolte, S. & Cordelieres, F. P. A guided tour into subcellular colocalization analysis in light microscopy. *J Microsc* **224**, 213-232, doi:10.1111/j.1365-2818.2006.01706.x (2006).

4 Rosenfeld, J., Capdevielle, J., Guillemot, J. C. & Ferrara, P. In-gel digestion of proteins for internal sequence analysis after one- or two-dimensional gel electrophoresis. *Analytical biochemistry* **203**, 173-179 (1992).

5 Guan, S., Price, J. C., Prusiner, S. B., Ghaemmaghami, S. & Burlingame, A. L. A data processing pipeline for mammalian proteome dynamics studies using stable isotope metabolic labeling. *Mol Cell Proteomics* **10**, M111 010728, doi:10.1074/mcp.M111.010728 (2011).

6 Clauser, K. R., Baker, P. & Burlingame, A. L. Role of accurate mass measurement (+/- 10 ppm) in protein identification strategies employing MS or MS/MS and database searching. *Anal Chem* **71**, 2871-2882 (1999).

7 Hasdemir, B. *et al.* Actin cytoskeleton-dependent regulation of corticotropin-releasing factor receptor heteromers. *Mol Biol Cell* **28**, 2386-2399, doi:10.1091/mbc.E16-11-0778 (2017).

8 Kozma, L. *et al.* The ras signaling pathway mimics insulin action on glucose transporter translocation. *Proc Natl Acad Sci U S A* **90**, 4460-4464, doi:10.1073/pnas.90.10.4460 (1993).
